# FishMORPH - An agent-based model to predict salmonid growth and distribution responses under natural and low flows

**DOI:** 10.1038/srep29414

**Published:** 2016-07-19

**Authors:** S. C. Phang, R. A. Stillman, J. Cucherousset, J. R. Britton, D. Roberts, W. R. C. Beaumont, R. E. Gozlan

**Affiliations:** 1 Department of Life and Environmental Sciences, Faculty of Science & Technology, Bournemouth University, Fern Barrow, Poole, Dorset BH12 5BB, UK; 2 CNRS, Université Paul Sabatier, ENFA; UMR 5174 EDB (Laboratoire Evolution & Diversité Biologique); 118 route de Narbonne, F-31062 Toulouse, France; 3 Université Toulouse 3 Paul Sabatier, CNRS; UMR5174 EDB, F-31062 Toulouse, France; 4 Game and Wildlife Conservation Trust, Salmon & Trout Research Centre, East Stoke, Dorset, UK; 5 Institut de Recherche pour le Développement, UMR BOREA IRD-MNHN-Université Pierre et Marie Curie, Muséum National d’Histoire Naturelle, 47 rue Cuvier, 75231 Paris cedex 5, France

## Abstract

Predicting fish responses to modified flow regimes is becoming central to fisheries management. In this study we present an agent-based model (ABM) to predict the growth and distribution of young-of-the-year (YOY) and one-year-old (1+) Atlantic salmon and brown trout in response to flow change during summer. A field study of a real population during both natural and low flow conditions provided the simulation environment and validation patterns. Virtual fish were realistic both in terms of bioenergetics and feeding. We tested alternative movement rules to replicate observed patterns of body mass, growth rates, stretch distribution and patch occupancy patterns. Notably, there was no calibration of the model. Virtual fish prioritising consumption rates before predator avoidance replicated observed growth and distribution patterns better than a purely maximising consumption rule. Stream conditions of low predation and harsh winters provide ecological justification for the selection of this behaviour during summer months. Overall, the model was able to predict distribution and growth patterns well across both natural and low flow regimes. The model can be used to support management of salmonids by predicting population responses to predicted flow impacts and associated habitat change.

Modified flow regimes can impact valuable freshwater fisheries and are likely to become an increasing threat due to greater water extraction, flow regulation and climate change driven modified temperature and precipitation patterns^1^,^2^,^3^. English chalk stream conditions of high invertebrate densities, low turbidity and favourable summer temperatures make them prime habitat for brown trout (*Salmo trutta*) and Atlantic salmon (*Salmo salar*)^4^,^5^,^6^ but their ecology is threatened by modified flows^7^,^8^. In the past, habitat association models have been commonly used to predict fish population responses to environmental change^9^; however, they are limited in their use in conditions outside of the ones for which they were parameterised^10^. Furthermore, this modelling approach does not account for fish behaviour as an adaptive response to an environmental change such as modified flow^11^. There is a need to pursue models capable of including an adaptive response in order to increase confidence in predicting fish population responses to habitat and support an evidence-based management approach^12^,^13^,^14^.

In fisheries management, two important population patterns are 1) how much fish grow and 2) how they distribute themselves across habitats. In temperate systems, fish growth is often greatest in productive summer months and can drive overwinter fish survival rates of fish whilst underpinning management stocking decisions^15^,^16^. In a modified flow environment, reduced flows can affect drift-feeding fish consumption rates and thus growth by altering flow velocities, depth and resource densities^17^. However, salmonids are territorial^18^,^19^ and individual fish moving in response to reduced flow will be limited by the availability of suitable areas for feeding^20^,^21^. The inclusion of behavioural processes is important for robust predictive models, as habitat and population patterns are driven by animal behaviour^11^,^22^. Models should also have the capacity to produce temporal and spatial site-specific population predictions.

One approach capable of linking behaviour to individual, population and environmental processes is individual-based ecology and can be implemented as Agent-Based/Individual-Based Models (ABM)^23^. MORPH is an ABM platform operating on optimal-foraging and fitness maximising principles; virtual ‘animals’ adapt their behaviours to feed or to rest based on bioenergetic rules, within virtual environments^24^ and has been successfully applied to coastal bird systems^25^,^26^. Elsewhere, non-MORPH salmonid ABMs have been developed and applied for fish populations in North America^27^.

In this study, we adapted the original MORPH ABM and assessed the ability of the model to predict salmonid somatic growth and distribution responses to flow changes. First, we recorded the environmental conditions and population patterns of a wild chalk stream salmonid population under natural flow (*NF*) and then modified, low-flow conditions (*MLF*). We then parameterised the agents in FishMORPH to have salmonid drift-feeding behaviour and bioenergetics using models obtained from literature. Appropriate habitat selection behaviours are needed to produce realistic patterns in spatially-explicit ABMs^28^ and we compared model results from alternative habitat selection models against the somatic growth and distribution of real salmonids under the two flows observed from the field study. The virtual fish habitat selection models tested were 1) random, 2) maximises consumption rates (*MCR*), 3) minimises predator-prey ratio (*MPR*), or 4) prioritises consumption rates over predator:prey ratios (*CR* > *PR*). The behaviour that most closely replicated the observed patterns was considered the most appropriate to be used in FishMORPH following a Pattern-Orientated Modelling approach (POM)^29^.

## Results

In this section we first describe the field study site and report the data collected from it. We then report the results comparing the growth and distribution from fish in the field study against the same pattern from virtual fish following the different movement rules.

### Field study

**Figure 1:**
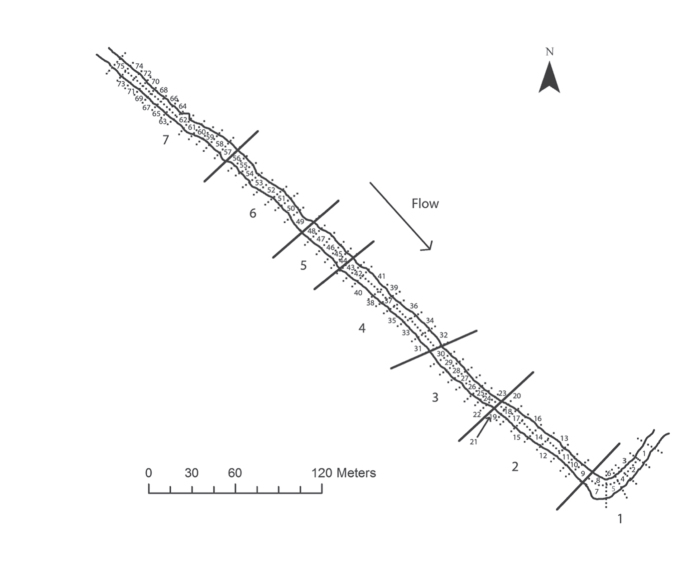
A schematic map of the study site, a section (520 m length; 6.25 m mean width) of chalk stream part of the River Frome, United-Kingdom (50° 40′44″N; 2° 0′42″ W) showing the two spatial resolutions i) 7 *stretches* (solid lines, large numbers, circa. 80 m in length) and the ii) 75 *patches* (dotted lines, small numbers, 39.6 ± 14.15 m^2^, mean ± SE).

Our study site was a section (520 m length; 6.25 m mean width) of the chalk stream part of the river Frome, United-Kingdom (50° 40′44″N; 2° 0′42″ W). We classified it at two spatial resolutions i) *stretches* (circa. 80 m in length) and ii) *patches* (39.6 ± 14.15 m^2^, mean ± SE) (Fig. 1). No barriers prevented fish movement. The study period began with *NF* conditions (17 July–23 September 2008, 68 days) after which we closed upstream river hatches to create *MLF* conditions (23 September–10 October 2008, 17 days). We recorded i) water temperature, ii) discharge and iii) daylight hours, and applied these to the entire study site as ‘global’ parameters in the model (see Supplementary Table S1). We also recorded i) predator densities and collected data on invertebrate densities to estimate ii) invertebrate prey abundance and applied these as stretch-specific parameters in the model (see Supplementary Table S2).

**Figure 2:**
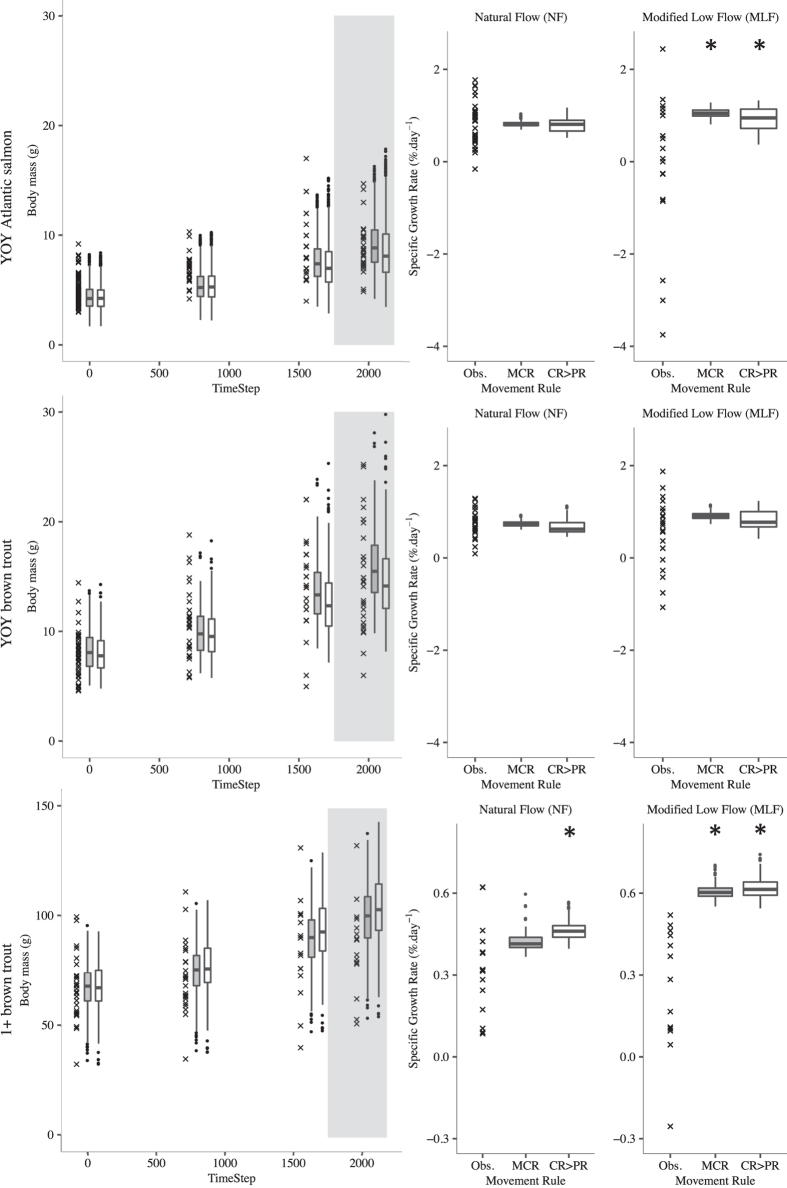
The body mass and specific growth rates of fish recorded in the field study (crosses) and predicted by models MCR (grey boxplots) and CR>PR (white boxplots). The fish were young-of-the-year (YOY) and one-year-old (1+) Atlantic salmon (*Salmo salar*) and brown trout (*Salmo trutta*). Plots in the left hand column show body mass and right hand for specific growth rates. For the body mass, each data point for field study results is an individual fish and model boxplots are from five replicate simulations. The white background indicates the period of natural flow and the grey background indicate the period of modified low flow. For specific growth rates (SGR), each data point for field study results is an individual recaptured PIT tagged fish and boxplots are of tagged fish in model from five replicate simulations. The stars indicate if the 95% quartile of differences between the movement rule model predicted and observed SGR from Bayesian statistics does not include zero.

We measured body mass and specific growth rates (SGR) from four electric fishing surveys. We caught a total of 1453 YOY Atlantic salmon, 283 YOY and 81 1+ brown trout of which 94, 96, and 79 were marked recaptures respectively. Over both flows, mean fish body mass increased and there was greater population variation in SGR during the *MLF* period (Fig. 2). YOY Atlantic salmon SGR showed the greatest pattern of change from the *NF* to *MLF* period while more YOY Atlantic salmon recorded slower growth rates in the latter period (Fig. 2).

### Validation of model predicted patterns

We then used the observed spatial and temporal patterns from the field study as the point of comparison for patterns generated from simulations of virtual fish following the four movement rules to validate the model.

### Pattern replication from 1) random and 3) MPR movement rules

Virtual fish that moved either *randomly* or *MPR* did not grow but consistently lost weight and had final distributions of very low body mass. These were not at all similar to observed body mass distribution patterns collected from field work and we removed *randomly* and *MPR* models from further analysis following the POM approach^29^.

### Body mass and specific growth rates pattern replication by 2) MCR and 4) CR>PR movement rules

We compared the body masses of *MCR* and *CR*>*PR* virtual fish against recorded body masses of real fish at time steps corresponding to when we performed fish surveys during the field study and found they were very similar across all three fish groups (Fig. 2). Both models produced similar median SGRs for each fish category and matched median observed SGRs well except for 1+ brown trout, where model SGRs were greater than those observed. Bayesian estimation of the probability of the difference between mean growth rates show that observed and virtual fish matched well for YOY brown trout across both flow periods, while YOY Atlantic salmon SGRs matched the *NF* period but not the *MLF* period (Fig. 2 and see Supplementary Table S3). Additionally, the model under predicted the observed variation in SGR in all cases.

### Stretch distribution pattern replication by 2) MCR and 4) CR>PR movement rules

**Figure 3:**
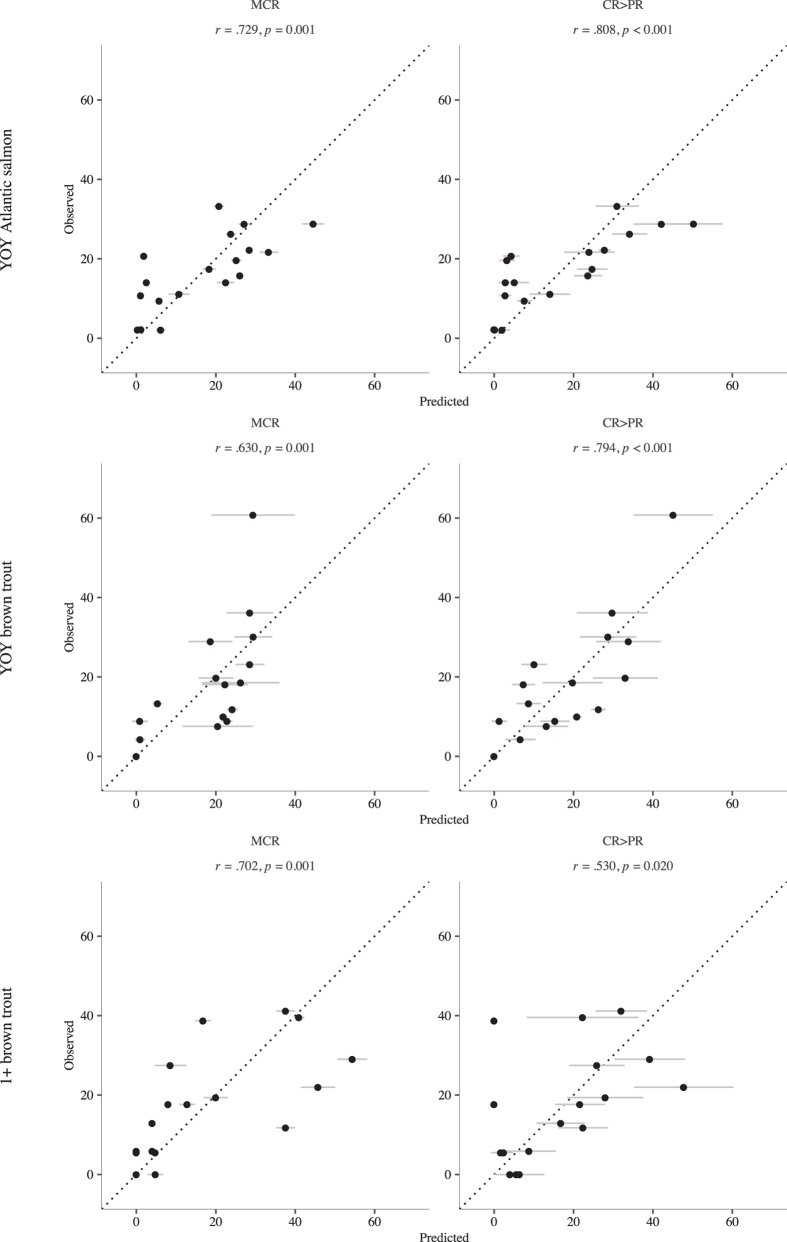
Observed vs. predicted fish numbers per stretch as percentage of total population by models MCR and CR>PR. Each point represents the mean and standard error of a stretch at 33, 68 (natural flow period) and 85 days (modified low flow) of the study period. Left-hand panels show data for the MCR models, right hand for CR>PR models. Rows correspond to fish categories as in Fig. 2.

We compared the stretch distributions predicted by *CR*>*PR* and *MCR* virtual fish with observed distributions^30^ at time points corresponding to when we performed the fish surveys during the field study and found they matched the observed distribution well across both flow regimes (Fig. 3). When pattern matching was analysed over both flow periods, *CR*>*PR* produced stretch distributions with higher Pearson’s *r* for YOY Atlantic salmon and YOY brown trout but not for 1+ brown trout, where *MCR* fish distributions were higher.

### Patch distribution pattern replication by 2) MCR and 4) CR>PR movement rules

**Figure 4:**
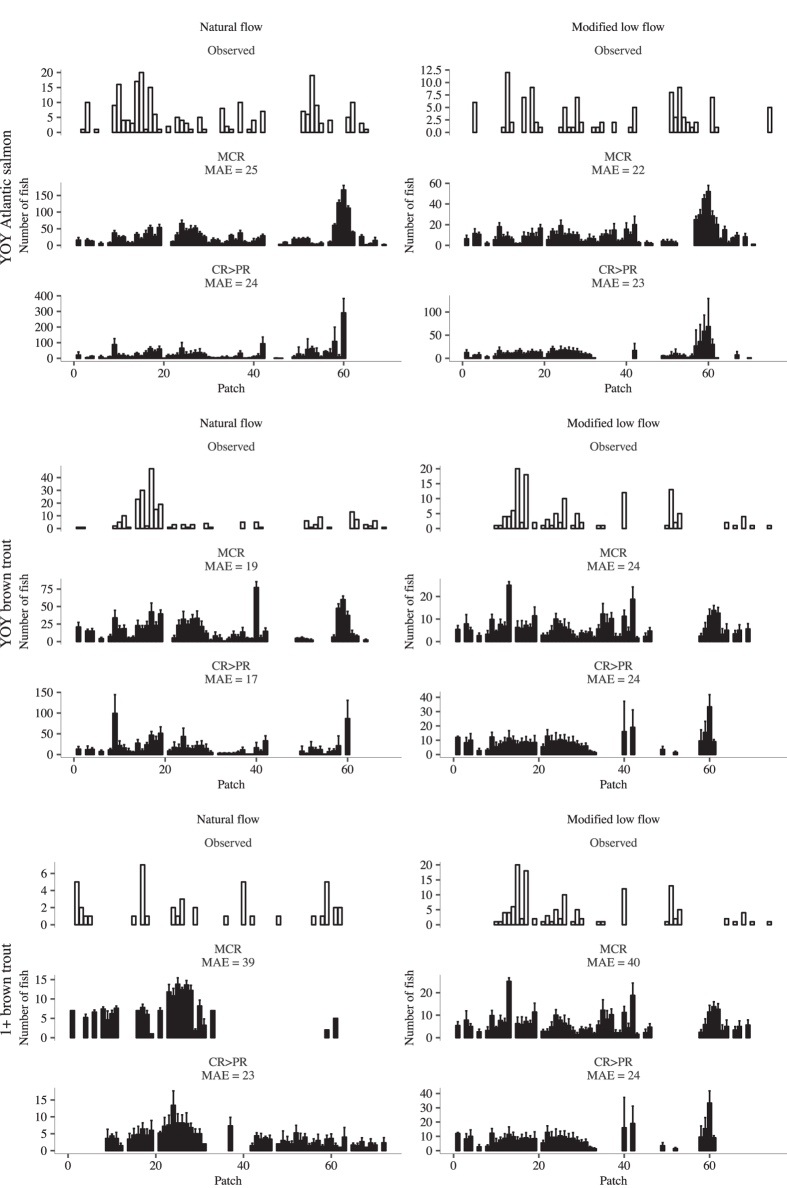
Number of PIT tagged fish per patch observed in the field study and by models MCR and CR>PR. Each bar point corresponds to one patch from 18 and 7 PIT tag tracking surveys during the natural flow and modified low periods. The bars show the mean and standard error from five replicate simulations at time steps corresponding to when the tracking surveys were performed in the field study. Gaps in the observed pattern indicate no fish were tracked in the patch in any of the surveys. Model standard error bars were obtained from five replicate simulations. White bars show the observed numbers and black bars are model predicted data. Left-hand panels show data for the natural flow period, right hand for the period of modified low flow. Rows correspond to fish categories as in Fig. 2.

We compared the patch occupancy distribution predicted by *CR*>*PR* and *MCR* virtual fish with observed distributions at time points corresponding to when the surveys were performed and found they were visually very similar (Fig. 4). In the patches where we tracked high numbers of fish the predicted patch densities are higher and vice versa. The Mean Absolute Error (MAE) between the observed and predicted was lower for the *CR*>*PR* movement rule for all distributions except for YOY Atlantic salmon *MLF* period. The difference between the two movement rules was greatest for 1+ brown trout. The fewer fish in the observed pattern was due to either tracking inefficiencies (estimated at ~40–50%^31^), tag loss or death by fish in the real study. However, the high number of sampling surveys (18 and 7 PIT tag tracking surveys during the *NF* and *MLF* periods respectively) provided a fairly robust overall pattern of the patches occupied or not occupied by fish.

### Proportion of time spent feeding predicited under 2) MCR and 4) CR>PR movement rules

**Figure 5:**
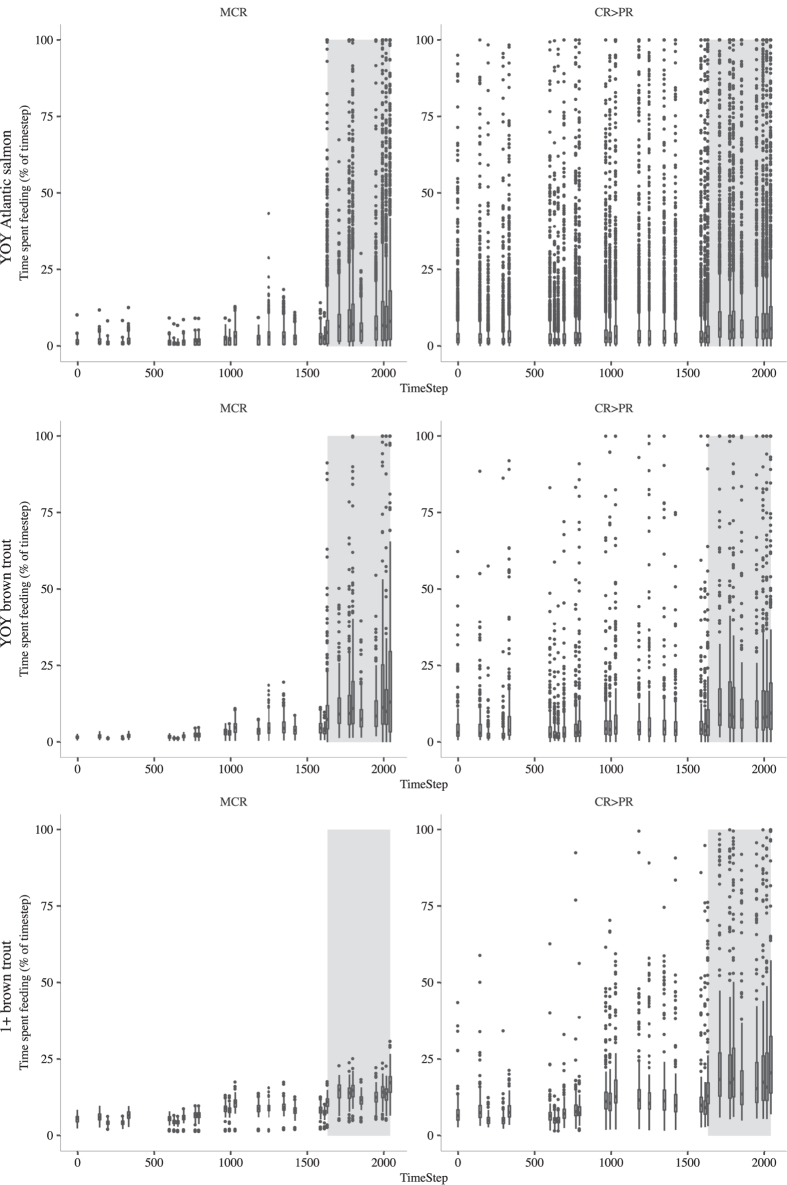
Time spent feeding by virtual fish at time steps in a simulation by models MCR and CR>PR. Each box plot with outliers is the combined distribution of individual fish in five replicate simulations and time steps correspond to when tracking surveys were performed in Fig. 3. The white background indicates the period of natural flow and the grey background indicates the period of modified low flow. Left-hand panels show data for the MCR models, right hand for CR>PR models. Rows correspond to fish categories as in Fig. 2.

We analysed the amount of time virtual fish moving under *MCR* and *CR*>*PR* movement rules spent feeding and found *MCR* fish fed for less time than *CR*>*PR* fish suggesting they reached the model defined time step maximum consumption threshold quicker (Fig. 5). Virtual fish feeding for the whole time step were likely not to reaching consumption maximum and so their overall growth rate would likely not be as high. The proportion of time spent feeding increased in the *MLF* period but the majority of fish still reached the threshold (i.e. less than 100% of the time spend feeding).

### Sensitivity analysis

Sensitivity analysis showed the model was most sensitive to parameters associated with bioenergetics whilst parameters pertaining to behavioural drift-feeding submodels had smaller effects (see Supplementary Table S4).

## Discussion

Robust predictions of the complex salmonid ecological response to altered flow regimes are needed for a proactive, evidence-based approach to fishery management. FishMORPH was able to predict body mass, growth rates (Fig. 2) and stretch and patch distribution (Figs 3 and 4) patterns of YOY and 1+ chalk stream Atlantic salmon and brown trout across two contrasting flow regimes. There was no calibration of unknown parameters and all patterns were independent from model construction: this strengthens the credibility of the model. The inclusion of adaptive behaviours increases predictive realism over other models where it is omitted. The *CR*>*PR* rule produced patterns most similar to the observed patterns, closely followed by the *MCR* movement rule, which performed less well in predicting distribution. The consistency in replicating distribution patterns across the two flow regimes supports the assumption of salmonid behavioural movement consistency across contrasting flows.

In our model, movement rules based solely on minimising predator:prey ratios (*MPR*) did not replicate patterns as well as consumption prioritising rules (*MCR* and *CR*>*PR*). The primary growth period for chalk stream salmonids is during the high productivity and thermally optimal summer and early autumn months and fish must maximise the potential growth for increased overwinter survival^32^,^33^ and reproductive success^34^,^35^. The lack of support for predator orientated movement rules may be due to low predation pressure at the study site. Whilst the density of the main piscivorous predator, European pike, in this study was not managed and can be considered natural, Atlantic salmon and brown trout are not preferred pike prey^36^ and other fish species were present. In addition, interactions in chalk streams may be limited for YOY salmonids as they associate with high-velocity habitats and ambush predators, such as pike, prefer lentic ones^37^. Future simulations could test the capacity of a consumption rate prioritising movement rule to predict growth and distribution patterns in habitats with higher rates of predator interactions and be used to comment on predator culling management regimes where reduced densities of large predators lead to increased densities of small ones^38^. Indeed, more patterns can be included for further testing of model assumptions and refinement of movement rules^28^,^39^ and in habitats beyond those simulated in this study^39^. *Random* and *MPR* movement rules produced unrealistic growth patterns and were removed from consideration but while ecologically unrealistic, the simulations were useful for testing the model setup^40^.

The model closely predicted mean observed body mass and growth rates of YOY brown trout for both *NF* and *MLF* periods. It better predicted these patterns in YOY Atlantic salmon during the *NF* period than in the *MLF* period where the model predicted high growth while the observed rates showed a decline. It is likely that the model predicted growth rates to remain high because the recorded thermal regime during the *MLF* period supported maximum growth^41^ and potential food consumption was not a limiting factor in the model as virtual fish were still able to reach their maximum consumption threshold in this period (Fig. 5). However, the *MLF* period of the field study coincided with the onset of autumn and possible altered energy budgets for overwintering and possible autumn smolt migration for anadromous Atlantic salmon^42^,^43^. The smoltification process occurs over several months and is a significant physiological and behavioural transformation requiring energy and can lead to a reduction of body condition^44^,^45^. In this system, Atlantic salmon parr most commonly undergo a spring migration but a proportion will also undertake a downstream migration phase in the autumn^42^; the timing of the *MLF* period in this study would have also been the period when any autumn migrating Atlantic salmon parr would have begun migration. Bioenergetic processes associated with overwintering and smolting were not included in this current model and it remains to be seen if including a more complex bioenergetic model would improve FishMORPH’s replication of the observed YOY Atlantic salmon growth pattern in the *MLF* period. It is also unknown whether the decline in observed growth is due to low flow conditions or overwintering/smolting processes.

The model was able to predict the increase in population SGR variation from the *NF* to *MLF* period but overall predicted variation was smaller than in the observed data. This could be a result of low intra-individual and inter-cohort variation between virtual fish in the model. Intra-individual variation in virtual fish was found in parameters of starting body mass, processing order, territorial size and predator vulnerability. While variation in body mass (i.e. fish size) would lead to slightly different inputs into feeding (e.g. capture area) and bioenergetic (e.g. Cmax and Rmax) submodels, the submodels themselves were the same for every fish. In reality, fish are likely to have individual differences in metabolism that would result in different growth rates before feeding back and leading to variation in behavioural responses^46^. A model that includes possible inter-individual variation may improve the match between predicted and observed distribution variance but a compromise with model complexity was made^47^. Additionally, behavioural variation including the time and bioenergetic cost of aggressive behaviours^48^, nocturnal feeding switching, prey switching and reproduction may improve predictions for 1+ fish.

Sensitivity analysis identified that the model is most sensitive to bioenergetic parameters, which is likely due to the productivity of the chalk stream environment. Prey density is very high in these stream environments with densities of 170,000 invertebrates.m^−2^ having been recorded in chalk streams^49^. At these densities, prey is not likely to be a limiting factor in chalk streams; maximum consumption limits and/or access resulting from territorial behaviour are important predictors of salmonid population ecology^21^, but ultimately prey will become a limiting factor at sufficiently low densities. Indeed, there is a negative relationship between salmonid territory size and prey densities^50^, which will affect the relative sensitivity of the model to the territory size parameter along a prey density gradient. This dynamic relationship is not currently present in FishMORPH as territory size is a fixed constant per virtual fish age group.

IBM validation is most commonly performed through pattern-orientated modelling, as more common statistical, empirical techniques applied in non-modelling situations are not always relevant because of lack of completeness of input parameters and inappropriate statistical techniques^51^. The undertaking of a field study specifically designed for model parameterisation and pattern collection provided relevant input data. Bayesian techniques, as used here, are a promising avenue of statistical inferences to measure pattern reproduction and can be used in other areas of IBM construction (e.g. parameterisation and calibration^52^. Statistical techniques were used to complement the POM validation philosophy; we discarded *Random* and *MPR* movement rules because they were unable to replicate body mass and growth patterns.

Predictions from a validated ABM can be used to investigate theoretical and applied questions^40^,^53^ and FishMORPH is able to replicate growth and distribution patterns from a real salmonid population responding to a changing flow regime. It can be used to predict the population response to fairly basic management questions (e.g. increasing the number of virtual fish to identify optimal stocking densities on growth). FishMORPH can also be used to investigate more complex and interactive relationships (e.g. magnitude of reduced flow and thermal regime warming) and across scales by altering multiple parameters operating (e.g. individual fish bioenergetics and reduced food densities). Finally, validated ABMs like FishMORPH can help support fishery management decisions responding to altered flow regimes and facilitate discussions about managing water with other water-use sectors.

## Methods

FishMORPH was parameterised and validated following a three-stage process; 1) a field study to collect input parameters and pattern data, 2) the construction of a bioenergetic and behaviourally realistic fish IBM within MORPH and 3) comparing predicted against observed patterns of growth and distribution (See Supplementary Figure S1).

### Ethics statement

All animal procedures followed strict guidelines set forward by the Home Office, UK and were performed within accordance with UK Home Office Regulations. The project was approved by the Bournemouth University ethics committee and was performed under the Home Office project licence number PPL 30/2626.

### Stage 1 – Field study

We recorded water temperature, discharge, daylight hours (sunrise and sunset), water height, channel gradient and aquatic vegetation patch cover across the study site (see Supplementary Methods). Drift and benthic invertebrate densities were collected from each stretch at dawn, midday and at dusk at monthly intervals, sorted into ten categories (1–3, 3–5, 5–7, 7–9 & 9–12 mm; aquatic or terrestrial in origin) and identified to family level. We accounted for the effect of clogging on drift estimation by drift net sampling^54^ by estimating drift density (invertebrates.m^−3^) from benthic invertebrate densities and drift net data to characterise its size distribution (see Supplementary Methods and Supplementary Table S5). There was a significant diel trend in drift densities in aquatically originating invertebrates (Kruskal-Wallis, *p* = 0.01) but not for terrestrial invertebrates (Kruskal-Wallis, *p* = 0.77). Thus, we parameterised the virtual environment to have aquatic invertebrates densities with intra-daily dynamics and terrestrial invertebrate densities that remained constant within a day. We found spatial and temporal differences in invertebrate densities across the study site and used linear interpolation between estimated densities to parameterise the invertebrate densities between sampling points (see Supplementary Methods).

We carried out a total of four fish surveys on 17^th^ July, 19^th^ August, 23^rd^ September and 10^th^ October 2008 (see^31^,^55^ and see Supplementary Methods). The number of Atlantic salmon and brown trout per stretch, fork length (*FL*, nearest mm), mass (*M*, nearest 0.1 g) and age (from scale samples) was recorded. A total of 91 YOY Atlantic salmon, 52 and 32 (YOY and 1+ respectively) brown trout were PIT tagged in the first survey. For each stretch and survey date, we estimated the number of fish and probability of capture^56^. Finally, we recorded the patch distribution of tagged fish by PIT-tag tracking surveys using a portable PIT detector^31^.

### Stage 2 – Model construction, virtualisation of the environment and initial population setup

The IBM was implemented using MORPH, an optimal-foraging IBM modelling platform^24^. Model description follows the Overview, Design, Details (ODD) protocol^57^,^58^. The model code and associated parameter files are available online (see Supplementary Data). The model’s virtual environment was defined to closely match the recorded field study conditions. The initial virtual fish population matched the measured size and distribution of the real fish at the start of the field study.

### Overview

#### Purpose

The aim was to model summer movement and growth of young-of-the-year (YOY) and one-year-old (1+) Atlantic salmon and brown trout under different discharge conditions using movement rules involving consumption rates and predator:prey ratio. Model validation was performed by comparing predicted patterns of body mass, growth and distribution against those collected from the field study.

#### Entities, state variables and scales

Simulations encompassed the *NF* and an *MLF* period of the field study and the model timestep was an hour. The virtual environment was a closed system with hierarchal spatial scales: i) global, ii) stretch and iii) patch. Global parameters applied across the entire environment with stretch and patch scales reflected parameters specific to their respective scales from the field study. Global variables were total duration (timesteps), day or night, water temperature (°C) and discharge (m^3^.s^−1^). The mass (dry mass, g) of invertebrates in the different invertebrate resource categories, their bioenergetic content (kJ.g^−1^) and the bioenergetic content of the fish (kJ.g^−1^) were kept constant (see Supplementary Table S6). Stretch variables were prey and predator densities (see Supplementary Table S2 and S5). Prey density (invertebrates.m^−3^) was defined into ten categories (1–3, 3–5, 5–7, 7–9 & 9–12 mm; aquatic or terrestrial in origin). Predator densities were modelled as a stretch parameter (no.m^−2^) and split into two size categories (*small,* FL<220 mm; *large,* FL>396 mm) to represent gape-limited predation on salmonids, no predators between the two thresholds were caught during the field study. Patch parameters were area (m^2^), location (in relation to other patches), the proportion of total area that is flowing (vs. slack water) (%), mean depth (m) and velocity (m.s^−1^) (see Supplementary Table S2).

Virtual fish were modelled as discrete individuals. Their constant state variables were species, age cohort, starting body mass, tagged status, starting stretch, territory size and diet. Fish diet was determined by fish length and prey-size diets. Dynamic state variables were current body mass (g) and location.

#### Variable processing and scheduling

FishMORPH processes variables in the order: 1) global, 2) stretch, 3) patch and 4) forager^24^ (see Supplementary Figure S2). Salmonid dominance is size-dependent^20^ and the model assumed size differences large enough to affect hierarchy were present between age cohorts but not within; 1+ fish were processed before YOY fish and fish in the same age cohort were processed in a random order.

Virtual fish had the following state variables parameterised: fork length (FL, mm), maximum consumption rate (C_max_, kJ.hr^−1^), maximum respiration rate (R_max_, kJ.hr^−1^), standard respiration rate (R_s_, kJ.hr^−1^), respiration cost of digestion (R_d_, kJ.hr^−1^), swimming cost whilst feeding (SC_feeding_, kJ.hr^−1^), resting (SC_resting_, kJ.hr^−1^), reaction distance (RD, m), maximum swimming velocity (V_max_, m.s^−1^), and capture area (CA, m^2^). Further prey consumption parameters were also modelled: encounter rate (RE, prey.hr^−1^), capture probability success (CPS, %), and capture rate (CR, prey.hr^−1^). Within a time step, fish were modelled as feeding or resting (or both) and selected the proportion returning the highest growth. Fish could only feed if there was sufficient feeding space. Bioenergetic equations of consumption and expenditure are used to calculate fish body mass at the end of each time step. This was then used as an input for calculating the changes during the next time step (see Supplementary Figure S3).

## Design concepts

The model’s *basic principle* was optimal-foraging theory^24^ with virtual fish fitness-maximising through adaptive behaviours. Distribution and growth patterns *emerged* from fish movement and feeding behaviour. Virtual fish *adapted* by i) moving between patches and ii) varying the time allocated to feeding and resting behaviour to maximise growth. Fish movement in a time step was limited to patches within a distance of one patch distance upstream or downstream. Virtual fish *sensed* patch variables within the movement distance. They *interacted* through competition for feeding space and the presence of other fish changed the patch predator:prey ratio that fish used in MPR and CR>PR movement rules. Sources of *stochasticity* were i) the starting body mass of virtual fish (drawn from a normal distribution with the mean ± SD calculated from the field study); ii) fish of the same age were processed in a random order and iii) a fish chose a patch at random if available patches returned the same measure of fitness. There were no *collectives* during a simulation as a virtual fish represented a single individual. *Observation* was through the creation of global, patch and virtual fish files at time steps that matched the times fish and PIT-tag surveys occurred during the field study.

### Details

#### Initialisation

The virtual environment was called in as external files. Each stretch started with the number of YOY Atlantic salmon, YOY brown trout and 1+ brown trout we collected from the first fish survey. Simulated fish had their initial starting mass drawn from a normal distribution with mean ± SD taken from stretch, age and species-specific field study recorded fish mass (see Supplementary Table S2).

#### Input data

In the external files, each state variable had a value for each time step. An external file also defined constant virtual fish state variables and the submodels that determined dynamic state variables.

#### Environment, fish bioenergetic and behavioural submodels

For reasons of space limitation, only factual descriptions of the processes essential for understanding movement behaviour and consumption are provided here. See Supplementary Methods for a complete description of the functions and justification.

#### Patch depth and water velocity

Patch depth and velocity were calculated for each time step using a quasi 1-D hydrological model that incorporated river discharge and accounted for spatial and temporal variability in the growth of *Ranunculus spp.*, the dominant aquatic macrophyte.

#### Resource density (10 categories) and energetic content

Mean dry biomass and energy density of collected invertebrates during the field study for each prey category was calculated using length-mass^59^,^60^,^61^ and mass-energy relationships^62^. Energy density of model invertebrates was set at the calculated mean of 22.13 kJ.g dry weight^−1^.

#### Feeding and territory

Virtual fish were only able to feed in flowing water. In order to feed, a fish had to be able to occupy a patch of flowing water equal to its territory size (TS). The TS of virtual fish was size and age dependent. For each patch, its ‘Available Feeding Area’ (AFA) was the area remaining after the total area of fish territories of feeding fish currently occupying it. Virtual fish were only able to *feed* in patches where the patch AFA exceeded their territory size, i.e. they were able to establish a feeding space. However, virtual fish were still allowed to occupy patches where AFA < TS but could only *rest*.

#### Gross Energy Intake (GEI) by an individual fish

Virtual fish were modelled as diurnal obligate, drift feeders. Virtual fish held a stationary position within the current by swimming at a speed equal to patch water velocity, and consumed drifting invertebrates passing through a ‘capture window’^28^,^63^. If a prey entered this window, a fish would swim towards it at its maximum swimming speed, attempt to capture it, before returning with the water current. The time taken for this was handling time (HT, s) and captures were not always successful with its probability of capture success (PCS, %) dependent on water velocity^64^. The capture window was square and a function of a fish’s reaction distance to a prey item, which in turn was related to its size^27^.

Bioenergetics was modelled as a function of energy intake and the costs of standard respiration, swimming activity and energy loss through faeces and urea. Energy consumption was maximally limited according to empirically measured consumption maximums^41^,^65^ (Cmax); if potential consumption rates allowed for energy intakes greater than this limit, fish would feed up to the limit and then spending the rest of the time step resting (or non-feeding). The energy cost of swimming was dependent on the activity of the fish with feeding swimming speeds equal to 100% of patch water velocity but dropping to 50% when it was resting^27^. Energy losses through faeces and urea was set at a constant 31%^41^,^65^. Growth was the net energy intake divided by the energetic content of the fish; the formulas used in the bioenergetic model were derived as gross efficiency and account for energy used to synthesise new tissue^66^.

#### Death

Virtual fish were removed from the system if their body mass fell below the conservatively set threshold of 1 g.

#### Predator density and predation risk

Virtual fish were modelled to be aware of predators with vulnerability being dependent on predator gape-size^67^; YOY salmonids were preyed on by both pike sizes, and considered the density of both small and larger predators in their movement, whilst 1+ fish only considered the density of large predator. Predation was incorporated as a predatory:prey ratio at the scale of the patch.

#### Stage 3 – Model simulation and validation

##### Simulation Experiments-Alternative habitat selection behaviours

Four different habitat selection behaviours were investigated:

1. *Random* – virtual fish moved randomly.
2. *Maximise consumption rate (MCR*) – virtual fish selected the patch that had the highest consumption rate.
3. *Minimise predation risk (MPR*) – virtual fish selected to the patch with the lowest predator:prey ratio.
4. *Prioritise maximum consumption rate then consider predation risk (CR*>*PR*) – Virtual fish only considered predation risk if the rate of consumption was equal or greater to *Cmax* and if so, selected the patch with the lowest predator:prey ratio. Otherwise, virtual fish moved to the patch with the highest consumption rate.

#### Model Variation

100 replicate simulations were run to measure between simulation variation of fish SGR (natural flow period, % body mass.day^−1^) and that five replicates allowed results to be within predicted ±0.06%, 0.05% and 0.01% for YOY Atlantic Salmon, YOY and 1+ brown trout respectively.

#### Model validation

Pattern-orientated validation^29^,^40^ was applied using time series data of: i) body mass, ii) specific-growth rates, distribution at the iii) stretch and iv) patch scale for YOY Atlantic salmon, YOY brown trout and 1+ brown trout. Model outputs were selected to match observations by date and location when data was collected relative to the study period. Bayesian estimation of the probability of the difference between observed and predicted growth rates were calculated using the R package BEST.R^68^.

#### Fish mass and specific growth rates

We compared observed and predicted fish body masses (g) at the time points when the last three fish surveys were performed. We also compared SGRs of real and virtual fish at the end of both the *NF* and *MLF* periods as: 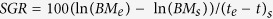
where *BM* = body mass (g), *t* = time (days) at the start (*s*) and end (*e*) between recaptures. Observed SGRs was calculated from recaptured, PIT tagged fish.

#### Stretch and patch distribution

We compared distribution at the scale of the stretch and the patch. For stretches, we calculated the percentage of the ‘total’ population located within each stretch, but removed stretches for the ‘total’ population where the probability of capture during the electric fishing surveys <0.2^56^.

We defined the observed patch distribution of real fish from the number of tagged fish recorded per patch from the PIT-tag tracking surveys. The distribution of virtual fish was the patch location of virtual fish that were also ‘tagged’ at time steps that corresponded to when the PIT tag tracking surveys were performed during the field study.

#### Sensitivity analysis

To test model sensitivity, parameters sourced from literature were adjusted (±5%) and predicted SGR were compared with the unadjusted model^26^.

## Additional Information

**How to cite this article**: Phang, S. C. *et al*. FishMORPH - An agent-based model to predict salmonid growth and distribution responses under natural and low flows. *Sci. Rep.* **6**, 29414; doi: 10.1038/srep29414 (2016).

## Supplementary Material

Supplementary Information 1

Supplementary Information 2
