# Supplementary material for: FishMORPH - An agent-based model to predict salmonid growth and distribution responses under natural and low flows
Source: Sci Rep. 2016 Jul 19;6:29414. doi: 10.1038/srep29414 (PMC4949470; doi:10.1038/srep29414)
Supplement: Supplementary Information 1 [file srep29414-s1.pdf]

Supplementary Information

FishMORPH - An agent-based model to predict salmonid growth and distribution responses under natural and low flows

Phang, S.C.[a,*,1] , Stillman, R.A.[a], Cucherousset, J.[b,c] , Britton, J.R.[a], Roberts, D.[d], Beaumont, W.R.C.[d] & Gozlan, R.E.[e]

[a] Department of Life and Environmental Sciences, Faculty of Science & Technology, Bournemouth University, Fern Barrow, Poole, Dorset BH12 5BB, UK

[b] CNRS, Université Paul Sabatier, ENFA ; UMR 5174 EDB (Laboratoire Evolution & Diversité Biologique) ; 118 route de Narbonne, F-31062 Toulouse, France.

[c] Université Toulouse 3 Paul Sabatier, CNRS ; UMR5174 EDB, F-31062 Toulouse, France.

[d] Game and Wildlife Conservation Trust, Salmon & Trout Research Centre, East Stoke, Dorset, UK.

[e] Institut de Recherche pour le Développement, UMR BOREA IRD-MNHN-Université Pierre et Marie Curie, Muséum National d'Histoire Naturelle, 47 rue Cuvier, 75231 Paris cedex 5, France

[*] Corresponding author at: Department of Evolution, Ecology, and Organismal Biology, The Ohio State University, 318 West 12th Avenue, Columbus, Ohio, 43210, USA. Tel: +1 614 2928088.

[1] Present address at: Department of Evolution, Ecology, and Organismal Biology, The Ohio State University, 318 West 12th Avenue, Columbus, Ohio, 43210, USA.

Supplementary Methods

Field study

In the field study, we measured the environment variables of: water temperature (Game and Wildlife Conservation Trust, East Stoke, UK, every 15 minutes), channel discharge (East Stoke Weir Station, Environment Agency, UK, every 15 minutes), water height and channel gradient (m, patch, Leica dGPS), running water and aquatic vegetation per patch (HABSCORE, 15th July, 28 August and 3rd October 2008).

We carried out invertebrate surveys on the 30th July, 28th August and 3rd October 2008 and i) drift and ii) benthos invertebrates at dawn, mid-day and dusk in each stretch. Drifting invertebrates were collected using drift nets (25 x 45 cm, 500µm mesh size) with the mouth tangential to river flow direction and bottom flush with the riverbed. The total duration of net submersion, water depth and velocity (measured at net mouth and ¼, ½ and ¾ of channel depth) were recorded for each sample collected. We sampled benthic invertebrates at the same time using surber nets (30 x 30cm, 500µm mesh size) and washed all substrate to a depth of one inch. We placed the surber nets near to but downstream of drift nets to minimise sampling interference and avoided locations that had previously been sampled earlier in the day. Collected invertebrates were preserved in a 70% industrial methylated spirit (IMS) solution and were classified to family level and body length measured (nearest 0.1mm).

We fished each stretch following a two-pass depletion electric fishing survey on the 17th July, 19th August, 23rd September and 10th October 2008 (see[52,53] for further details). The numbers of Atlantic salmon and brown trout and for each fish, we measured fork length (*FL*, nearest mm), mass (*M*, nearest 0.1 g) and age (from scale samples). We imposed a minimum tagging size of *FL* > 60mm. We estimated the number of YOY Atlantic salmon, YOY brown trout and 1+ brown trout ($\hat{N}$) in a stretch by: $\hat{N} = C_1^2 \left(C_1 - C_2 \right)$. The probability capture (*p*) per stretch was

calculated by: $p = (C_1 - C_2)/C_1$, where C_1 and C_2 = number of fish caught first and second passes respectively[54]. We tracked the distribution of tagged fish at the patch scale by PIT-tag tracking surveys using a portable PIT detector (*UF, n*=18*; MLF,* n=7; see [53]).

FishMORPH environmental submodels

Water temperature and run per patch

Water temperature (every 15 mins) was converted into hourly means. The percentage 'run' per patch in the *UF* and *MLF* periods was recorded during HABSCORE surveys. These remained constant for timesteps within the respective periods.

Flow depth and velocity

We used riverbed gradient, water depth, discharge data and aquatic vegetation cover to calibrate a 1-D flow model to calculate patch parameters of mean velocity and depth. Hourly estimates of channel discharge were the mean of discharge data (every 15 mins). The hydrological submodel was based on a step backwater solution of the 1-D gradually varied flow equations[73], which can be written as:

$$\frac{dh}{dx} = \frac{SL_0 - SL_f}{1 - Fr^2}$$

where h = flow depth, x = distance along the downstream channel axis, Fr is the Froude number, SL_0 is the bed slope and SL_f is the friction slope, estimated using the Manning resistance law:

$$\frac{V^2 n^2}{h^{4/3}} = \left(\frac{Q}{W}\right)^2 \times \left(\frac{n}{h^{5/3}}\right)^2$$

where V = flow velocity, Q = discharge, W = channel width and n = Manning friction coefficient (quadratic of % aquatic vegetation cover).

We defined values of *n* as a quadratic function of the percentage of the riverbed covered by vegetation within each patch. This relationship was calibrated using measurements of percentage vegetation, flow depth and velocity made throughout the study reach at a known discharge. This approach is termed quasi 1-D because a uniform value of S_f is assumed at each stretch. This uniform value was determined by integrating over the distribution of depths and n values at each section (note that some sections contain two patches and in each patch a constant value of *n* is assumed).

Invertebrate densities

We classified collected invertebrates into ten categories depending on size (5 size categories; 1-3; 3-5; 5-7; 7-9; 9-12 mm) and origin (aquatic vs. terrestrial). We assumed invertebrate densities (number of invertebrates. m^3) to be different between stretches but not between patches found in the same stretch. The amount of invertebrate prey available per patch to a virtual fish was a function of the patch's flow parameters of depth and velocity and the virtual fish's feeding submodels.

Estimating drifting invertebrate densities from drift net collection may be erroneous errors due to processes like net clogging during data collection[51] Instead, we parameterised the invertebrate drift within the virtual environment using benthic invertebrate densities to estimate drift densities with a size structure taken from drift samples. We assumed the sampling biases from drift net sampling were constant for all invertebrate sizes and the size structure of the invertebrate drift was represented in the drift net data.

At all moments, a percentage of the benthos invertebrate population enter the water column and 'drift'[74,75]. The percentage of 'spontaneous benthos drift' (SBD) has been measured to range from 0.004% to 0.13% of the total benthos population[75,76]. We used the higher estimate to reflect the high productivity of chalk streams. The drift density originating from benthos invertebrates ($DD_{benthos}$, number.m^{-3}) was calculated by:

$$DD_{Benthos} = \frac{SBD \times BD_s \times SA_s}{\sum \left(PA_s \times PD_s \right)}$$

where s = stretch, SBD = spontaneous benthos drift (0.0013 from [75]), BD = benthos density from surber nets, SA = stretch area (m^2), PA = patch area, PD = patch depth. We then applied the size structure ratio derived from drift net samples to this density to estimate invertebrate drift densities (dawn, mid-day and dusk) for each size category at each sample date (Supplementary Table S1 and Supplementary Data Online).

Estimating daily and diel drift densities between samples dates

We used linear interpolation to estimate invertebrate drift densities at dawn, mid-day and dusk for each day between sample dates. We also used a linear interpolation between these points to create the diel dynamics in drift densities (aquatic in origin) with densities at night assumed to be the same as dusk densities[5]. We found no significant trend in the drift densities of terrestrial invertebrate drift in collected samples (Kruskal-Wallis, $p>0.05$) and we fixed densities to be constant throughout the daylight hours and zero at night.

Feeding space and territory establishment

Two conditions had to be met for virtual fish in FishMORPH to successfully feed. The first was that they were only able to feed in flowing water (i.e. in the patch's run as opposed to slack water) and secondly, they were also required to establish a territory (*Territory Area*, m^2) within this area. The area of flowing water within a patch was calculated from the patch parameters of run (% of total area) and patch area (m^2). The *Available Feeding Area* (AFA, m^2) was the area of flowing water remaining after the territory of all fish that were already present and were feeding. This was calculated by:

$$AFA_{p,t} = R_p \times A_p - \sum\left(F_{p,t} \times TA_a\right)$$

where p = patch, R = run (% of patch), A = area (m^2); F = the number of feeding fish; TA = Territory Area (m^2); t = timestep and a = the age of the feeding fish. Virtual fish were only able to feed within a patch if the AFA within the patch could accommodate the fish's territory ($AFA > TA$). Virtual fish were able to occupy a patch and not *feed* (i.e. *rest*).

Gross Energy Intake (GEI) by virtual fish

Virtual fish in FishMORPH were modelled with salmonid drift feed behaviour; to hold a stationary position in flowing water by swimming at a speed constant to the velocity of the water[27] and consuming drifting invertebrates which pass through a 'capture window'[61]. The drift-feeding submodel of virtual fish considered: (i) the availability and density of invertebrate prey; (ii) the detection of prey items entering a 'capture window'; and (iii) the probability of its successful capture[27,70]. The maximum distance a virtual fish could detect a drifting invertebrate was its *Reaction Distance* (*RD*). A virtual fish's *Capture Area* (*CA*) is a rectangle based on this distance. If the depth of water within a patch was less than the reaction distance, *RD* was replaced

by water height[27]. Virtual fish were modelled as diurnal visual feeders[61] by making $RD = 0$ at night. The number of drift invertebrates passing through this capture window, the *Encounter Rate* (*ER*, items.hr^{-1}) was calculated by:

$$ER_f = 3600 \times DD_f \times V_{p,t} \times CA_f$$

where f = fish, 3600 = number of seconds in one hour; *DD* = density of drifting invertebrates part of the fish's diet (m^3), *V* = water velocity (m.s^{-1}), *CA* = capture area (m^2), *p* = patch, *t* = timestep.

Virtual fish captured drifting invertebrates by first detecting it at its *RD*, then swimming to it at maximum swimming velocity, capturing it and then returning to its initial location with the flow of the water. The time taken to complete this sequence was its *Handling Time* (*HT,* hour) for each encounter[61]:

$$3600 \times \frac{1}{2} \times RD \times \left(\frac{1}{V_{\max}} + \frac{1}{Patch_{Velocity}} \right)$$

where *RD* = reaction distance (m), *MSV* = maximum swimming velocity (m.s^{-1}), *V* = water velocity (m.s^{-1}), *f* = the virtual fish, *p* = patch and *t* = timestep.

Virtual fish were not always successful in capturing drifting invertebrate prey. The probability of a successful capture (*Capture Probability Success, CPS, %*) had a negative relationship with water velocity[62]. The total number of successful captures in a hour of drifting invertebrates in each size category was a virtual fish's *Capture Rate (*C_{rate}, invertebrates.hr^{-1}). This followed a Type II Holling's disk function and was calculated as:

$$CR_{i,t} = CPS \times ER \Big/ \left(1 + \sum_{i}^{10} CPS \times ER_{i,t} \times HT \right)$$

where *i* = invertebrate size category; *t* = time step; *CPS* is capture probability of success from Piccolo et al. 2008; *ER* is the encounter rate (items.hr^{-1}); *HT* is the handling time (hr). The *Total*

Capture Rate (*TCR,* inverterbrates.hr^{-1}) for drifting invertebrate prey sizes was the sum of the *CR* for each size group: $TCR_t = \sum_{1}^{10} CR_{i,t}$.

Maximum consumption (C_{max})

Salmonids have a bioenergetic *Maximum Consumption* (C_{max}) threshold[68] and FishMORPH included this for realism and was calculated by:

$$C_{\max.kJ} = 0.00416868 \times C_{\max.Cal} / DL$$

where $C_{max.kJ}$ = maximum consumption (kJ.hr^{-1}), $C_{max.Cal}$ = maximum daily consumption[68] (calories.day^{-1}) and *DL* = number of daylight hours in the day.

Total Consumption

FishMORPH calculated the total number of invertebrate prey consumed by a virtual fish in a timestep or its *Total Consumption* (*TC,* invertebrate.hr^{-1}) by the relationship between *Total Consumption Rate* (*TCR*) and *Maximum Consumption* (C_{max}) threshold:

$$TC_t = \begin{cases} C_{\max.kJ} / E_i & \text{if } TCR_t > \left(C_{\max.kJ} / E_i\right) \\ TCR_t & \text{if } TCR_t \le \left(C_{\max.kJ} / E\right) \end{cases}$$

where *TCR* = *Total Consumption Rate* (invertebrates.hr^{-1}), $C_{max.kJ}$ = *Maximum Consumption* threshold (kJ.hr^{-1}); E_i = energetic content of invertebrates (kJ.invertebrate^{-1}). The total consumption in energy (TC_{kJ}, kJ.hr^{-1}) was calculated by multiplying *TC* by the invertebrate energy density: $TC_{kJ,t} = TC_t \times E_t$.

Proportion of time feeding per timestep

Virtual fish were only able to feed if the time step was during daylight hours and if there was sufficient Available Feeding Space (AFS, m^2) in the patch. If both conditions were met, the

virtual fish's activity (feeding, $A_{feeding}$ or resting, $A_{resting}$, %.timestep^{-1}) in a timestep was calculated by:

$$A_{feeding,t} = \begin{cases} 100 \times C_{\max.kJ} / E_i & \text{if } TCR_t > \left(C_{\max.kJ} / E_i\right) \\ 100 & \text{if } TCR_t \leq \left(C_{\max.kJ} / E\right) \end{cases}$$

where TCR = total capture rate of drifting invertebrates (invertebrates.hr^{-1}), t = timestep; C_{max} = *Maximum Consumption* threshold (kJ.hr^{-1}), E_i = the energetic content of invertebrates (kJ.invertebrate^{-1}). Consequently, the time spent resting was: $A_{resting,t} = 1 - A_{feeding,t}$.

Faeces and Urea

Brown trout lose consumed energy through faeces (F) and urea (U). This loss was measured to be 30-32% of consumed energy across a variation of bodymass and temperature[68]. FishMORPH fixes to loss of consumed energy to these processes at 31% by setting the assimilation efficiency, or the conversion of consumed energy into bioenergetically available energy to a constant 69% at all levels of consumption[61].

Respiration (R)

The non-swimming bioenergetic costs associated respiration processes (R_{max}), standard metabolism ($R_{standard}$), digestion ($R_{digestion}$) and activity ($R_{activity}$) for brown trout are dependent on body mass, digestion temperature[68]. Virtual fish respiration in FishMORPH occurs at every timestep and was calculated by:

$$R_{\max.kJ,t} = 0.0041868 \times R_{\max.Cal} / 24$$

where $R_{max.kJ}$ = the energy associated with respiration processes in a timestep (kJ.hr^{-1}), t = time step and $R_{max.Cal}$ = the energy associated with respiration processes in a day (calories.day^{-1}) from [68]. FishMORPH considers the respiration associated with non-swimming activity ($R_{activity}$) to be negligible in the equations of R_{max}[61,68]. To limit complexity, it also considers the respiration

energy associated with digestion activities ($R_{digestion}$) to be constant even under consumption rates below maximum.

Swimming costs (SC)

Virtual fish in FishMORPH swam at 100% and at 50% of patch velocity when feeding and resting respectively[61,63,68]. The total bioenergetic costs of swimming per time step or *Total Swimming Cost* (T*SC,* kJ) was calculated by:

$$TSC_t = A_{feeding,t} \times \left(SC_{feeding,t}\right) + A_{resting,t} \times (SC_{resting,t})$$

where t = timestep, $A_{feeding}$ = time spent feeding (%), $A_{resting}$ = time spent resting (%), $SC_{feeding}$ = bioenergetic cost of swimming (kJ.hr^{-1}) while feeding and $SC_{resting}$ = bioenergetic cost of swimming (kJ.hr^{-1}) while resting for that time step in that patch.

Net energy intake (NEI)

FishMORPH calculates the net body mass (ΔB, g) change per time step by:

$$\Delta B = \left(TC_{kJ,t} \times \left(1 - FU\right) - R_{\max,t} - TSC_t\right) / E_f$$

where FU = fixed energy lost in faeces and urea (%), TC_{kJ} = total consumed energy (kJ), R_{max} = total energy lost through respiration (kJ), SC = the swimming cost (kJ.hr^{-1}), t = time step, and E_f = the specific energy density for virtual fish (kJ.g^{-1}).

Death

Modelling starvation-induced mortality involves is complex and involves dynamic factors including but not limited to: body mass composition, bioenergetic expenditure, temperature and age[77]. FishMORPH adopts a simple starvation threshold where virtual fish die if their body mass decreased to a conservatively set threshold of 1g.

Predator density and predation risk

Prey fish alter their behaviours in the presence of predators[78–80]. Virtual fish in FishMORPH were modelled to be aware of densities of the Northern pike (*Esox lucius*), primary piscivorous aquatic predator. Pike were classified into *small* (fork length < 218mm) and *large* (fork length > 396mm) and we did not catch any pike between the thresholds during the field study. Number of pikes per size group was transformed into a patch parameter by dividing the number of pike by the total area of the stretch. The density (pike.m^2) was a fixed constant per patch. Pike gape size was calculated at 29mm and 52mm for small and large pike respectively[65]. YOY virtual fish were small enough to be potentially predated by both sizes of pike and thus considered the density of both predator groups in their movement. 1+ virtual fish were wide enough to exclude small pike as predators and thus they only considered the density of large pike in their movement. Virtual fish considered the Predator:Prey ratio (*PPR*) by:

$$PPR_p = \begin{cases} \left(LPD_p + SPD_p\right) \big/ \sum F_{YOY.p} & \text{if Age}_f = YOY \\ LPD_p \big/ \left(\sum F_{YOY.p} + \sum F_{1+,p}\right) & \text{if Age}_f = 1+ \end{cases}$$

where p = patch, f = fish, *LPD* = density of large pike (large pike.m^2), *SPD* = density of small pike (small pike.m^2), *F* = fish per age class, *YOY* = young-of-the-year fish, *all* = YOY + one-year-old fish and age = the age of the fish.

Supplementary Figures

Supplementary Figure S1. The study followed three stages to quantitatively validate FishMORPH. The first stage involved exposing a real salmonid population to a change in flow and recording both the environmental conditions and population responses. FishMORPH was then initialised with a virtual fish population closely matching the population composition and body mass of real fish as recorded at the start of the field study. Virtual fish were then simulated with a virtual environment that replicated the field study environment. Finally, the growth and spatial patterns of virtual fish within FishMORPH was compared with the same patterns of real fish as validation.

Supplementary Figure S2. Each timestep, FishMORPH performs processes in a hierarchal sequence and beginning with calculating environmental variables either from submodels or input files starting with global, stretch then patch parameters. After this, it moves onto processing individual virtual fish beginning with the oldest fish group.

Supplementary Figure S3. Virtual fish in FishMORPH adapt their behaviours to maximise growth. They select from feeding or resting (i.e. not feeding) based on patch parameters and the net bioenergetic result of the behaviours.

Supplementary Tables

Supplementary Table S1. The global parameters used to define the model environment. Parameter values are those recorded in the field study (mean ± SD shown).

Name	Unit	Value
Simulated period	days	85
- natural flow		68 (17th Jul – 23rd Sep)
- modified low flow		17 (23rd Sep – 10th Oct)
Time steps	hour	2040
Mean daylight	$hours.day^{-1}$	13.72 ± 1.47
Mean water temperature	°C	
- natural flow		15.47 ± 1.49
- modified low flow		12.29 ± 0.86
Mean discharge	$m^3.s^{-1}$	
- natural flow		0.778 ± 0.207
- modified low flow		0.381 ± 0.046
Invertebrate (prey) energy density	$kJ.g.dw^{-1}$	22.13
Invertebrate biomass	dw.mg	
Aquatic		
A1 : 1-3mm		0.10
A2 : 3-5mm		0.32
A3 : 5-7mm		1.09
A4 : 7-9mm		2.41
A5 : 9-12mm		5.92
Terrestrial		
T1 : 1-3mm		0.25
T2 : 3-5mm		1.23
T3 : 5-7mm		3.43
T4 : 7-9mm		6.85
T5 : 9-12mm		7.14

Supplementary Table S2. Summarised habitat, predator and virtual fish parameters defining the virtual environment at the scale of the stretch. The virtual fish were young-of-the-year (YOY) and one-year-old (1+) Atlantic salmon (*Salmo salar*) and brown trout (*Salmo trutta*). The virtual environment matched the chalk stream study site under the two flow regimes of natural flow (romanised) and modified low flow (italicised) (mean ± SD shown).

Parameter	Stretch						
	1	2	3	4	5	6	7
Area (m^2)	328.5	448.4	484.5	422.6	288.5	333.8	666.6
Patches							
- number	8	12	11	11	6	8	19
- area(m^2)	41.1±23.0	37.4±13.5	44.0±12.5	38.4±19.8	48.1±11.1	41.7±11.1	35.1±8.6
- run (%)	44.4±44.4	74.7±28.1	84.1±17.1	59.6±37.6	42.5±27.7	79.1±23.9	67.6±25.7
	41.2±44.4	*73.0±32.8*	*59.5±37.6*	*65.5±31.4*	*51.7±42.2*	*80.0±29.8*	*68.2±28.2*
Water							
- velocity	0.33±0.05	0.33±0.06	0.32±0.06	0.25±0.06	0.20±0.04	0.26±0.04	0.33±0.10
(m.s^{-1})	*0.23±0.04*	*0.26±0.05*	*0.25±0.04*	*0.19±0.05*	*0.14±0.03*	*0.20±0.02*	*0.26±0.10*
- depth	0.30±0.05	0.27±0.05	0.29±0.07	0.38±0.08	0.52±0.08	0.42±0.06	0.31±0.10
(m)	*0.23±0.03*	*0.18±0.03*	*0.22±0.05*	*0.28±0.07*	*0.42±0.07*	*0.32±0.04*	*0.23±0.08*
Predator density							
- small	0.002	0.001	0.003	0.001	0.008	0.001	0.000
(no.m^2)	*0.002*	*0.000*	*0.000*	*0.005*	*0.019*	*0.001*	*0.001*
- large	0.002	0.001	0.002	0.002	0.012	0.002	0.003
(no.m^2)	*0.003*	*0.000*	*0.000*	*0.001*	0.000	0.000	*0.000*
Starting number and body mass (mean ± SE; g)							
- YOY Atlantic salmon	8	106	95	29	1	26	70
	3.43±0.78	3.81±0.89	4.27±1.07	4.44±1.17	3.30	5.03±1.00	4.86±1.31
- YOY brown trout	3	21	8	4	0	4	6
	6.33±0.70	8.20±1.84	7.71±0.85	9.73±2.34	-	7.80±2.05	7.57±1.17

	8	5	7	4	0	0	1
- 1+ brown trout	68.89±5.40	62.60±13.62	73.56±10.51	73.74±12.12	-	-	65.96

Supplementary Table S3. Observed and FishMORPH predicted specific growth rates (SGR) of young-of-the-year (YOY) and one-year-old (1+) Atlantic salmon (*Salmo salar*) and brown trout (*Salmo trutta*) during the natural flow (*NF*) and modified low flow (*MLF*) periods. The simulated movement behaviour of virtual fish were: maximise consumption rate (MCR) and prioritise consumption rate then consider predation risk (CR > PR). The movement behaviour that produced the smallest mean difference between the observed and predicted SGR and if the 95% quartile of differences from Bayesian statistics straddles zero are highlighted in bold.

Fish	Flow regime	Movement rule	SGR (Mean ± S.D.)	Difference from real fish	
				Mean	95% HDI (lower, upper)
YOY Atlantic salmon	NF	Observed	0.80 ± 0.47	-	-
		MCR	0.83 ± 0.06	0.04	**-0.21, 0.13**
		CR > PR	0.77 ± 0.14	**0.03**	**-0.13, 0.20**
	MLF	Observed	0.02 ± 1.52	-	-
		MCR	1.07 ± 0.11	1.14	1.97, 0.32
		CR > PR	0.90 ± 0.22	**1.00**	1.83, 0.16
YOY brown trout	NF	Observed	0.77 ± 0.29	-	-
		MCR	0.73 ± 0.06	**0.03**	**-0.08, 0.15**
		CR > PR	0.66 ± 0.13	0.12	**-0.00, 0.23**
	MLF	Observed	0.60 ± 0.70	-	-
		MCR	0.91 ± 0.07	0.33	**0.64, 0.00**
		CR > PR	0.82 ± 0.18	**0.24**	**-0.56, 0.08**
1+ brown trout	NF	Observed	0.32 ± 0.18	-	-
		MCR	0.42 ± 0.02	**0.10**	**0.19, 0.00**
		CR > PR	0.46 ± 0.04	0.14	0.04, 0.39

	Observed	0.26 ± 0.23	-	-
MLF	MCR	0.60 ± 0.02	**0.34**	0.49, 0.19
	CR > PR	0.62 ± 0.03	0.35	0.50, 0.20

Supplementary Table S4. The effect of a ± 5% change in field collected and literature sourced parameters on mean ± SD virtual fish specific growth rates (*SGR*, %.day^{-1}) during the natural flow period.

Fish	Parameter	Mean ± SD SGR<br>-5% Parameter change	 -5% Parameter change
YOY Atlantic salmon	Maximum Consumption *(Cmax)*	0.202±0.125	1.170±0.223
	Energy lost through faeces and urea (*FU)*	0.465±0.146	0.968±0.167
	Respiration costs of digestion ($R_{digestion}$)	0.680±0.168	0.811±0.163
	Drifting invertebrate density	0.685±0.175	0.751±0.131
	Feeding Metabolic Rate ($MR_{feeding}$)	0.720±0.147	0.744±0.154
	Invertebrate specific energy density	0.726±0.174	0.762±0.164
	Reaction Distance (*RD*)	0.727±0.142	0.765±0.182
	Handling Time (*HT*)	0.729±0.156	0.767±0.154
	Capture Area (*CA*)	0.732±0.140	0.759±0.156
	1+ fish Territory Size	0.734±0.152	0.780±0.175
	Swimming bioenergetic cost while resting ($SC_{Resting}$)	0.735±0.172	0.687±0.153
	Capture Probability Success (*CPS*)	0.737±0.131	0.723±0.135
	Maximum Swimming Velocity (*MSV*)	0.738±0.152	0.743±0.157
	Swimming bioenergetic cost while feeding ($SC_{feeding}$)	0.752±0.147	0.738±0.165
	Fish specific energy density	0.757±0.172	0.688±0.164
	YOY fish Territory Size	0.765±0.198	0.750±0.156
	Standard respiration rate ($R_{standard}$)	0.864±0.159	0.603±0.162
	Resting Metabolic Rate ($MR_{resting}$)	0.914±0.161	0.524±0.169
	Maximum Respiration (R_{max})	0.914±0.173	0.529±0.161
YOY brown trout	Maximum Consumption *(Cmax)*	0.171±0.104	1.170±0.223
	Energy lost through faeces and urea (*FU)*	0.400±0.123	0.968±0.167
	Respiration costs of digestion ($R_{digestion}$)	0.569±0.134	0.811±0.163
	Drifting invertebrate density	0.600±0.149	0.751±0.131
	Invertebrate specific energy density	0.613±0.148	0.762±0.164
	Feeding Metabolic Rate ($MR_{feeding}$)	0.625±0.124	0.744±0.154
	1+ fish Territory Size	0.629±0.121	0.780±0.175
	Swimming bioenergetic cost while resting ($SC_{Resting}$)	0.630±0.147	0.687±0.153
	Reaction Distance (*RD*)	0.633±0.121	0.765±0.182
	Capture Area (*CA*)	0.634±0.130	0.759±0.156
	Maximum Swimming Velocity (*MSV*)	0.634±0.131	0.743±0.157

	Capture Probability Success (*CPS*)	0.641±0.118	0.723±0.135
	Handling Time (*HT*)	0.641±0.139	0.767±0.154
	YOY fish Territory Size	0.642±0.169	0.750±0.156
	Swimming bioenergetic cost while feeding ($SC_{feeding}$)	0.651±0.140	0.738±0.165
	Fish specific energy density	0.652±0.156	0.688±0.164
	Standard respiration rate ($R_{standard}$)	0.746±0.142	0.603±0.162
	Maximum Respiration (R_{max})	0.784±0.141	0.529±0.161
	Resting Metabolic Rate ($MR_{resting}$)	0.798±0.145	0.524±0.169
1+ brown trout	Maximum Consumption *(Cmax)*	0.147±0.031	1.170±0.223
	Energy lost through faeces and urea (*FU)*	0.301±0.032	0.968±0.167
	Respiration costs of digestion ($R_{digestion}$)	0.419±0.034	0.811±0.163
	Reaction Distance (*RD*)	0.449±-.033	0.765±0.182
	Capture Area (*CA*)	0.450±0.032	0.759±0.156
	Drifting invertebrate density	0.452±0.032	0.751±0.131
	Handling Time (*HT*)	0.453±0.034	0.767±0.154
	Maximum Swimming Velocity (*MSV*)	0.453±0.035	0.743±0.157
	Capture Probability Success (*CPS*)	0.454±0.034	0.723±0.135
	Invertebrate specific energy density	0.454±0.036	0.762±0.164
	YOY fish Territory Size	0.458±0.032	0.750±0.156
	Feeding Metabolic Rate ($MR_{feeding}$)	0.460±0.035	0.744±0.154
	1+ fish Territory Size	0.460±0.035	0.780±0.15
	Swimming bioenergetic cost while feeding ($SC_{feeding}$)	0.462±0.036	0.738±0.165
	Swimming bioenergetic cost while resting ($SC_{Resting}$)	0.472±0.039	0.687±0.153
	Fish specific energy density	0.479±0.035	0.688±0.164
	Standard respiration rate ($R_{standard}$)	0.539±0.035	0.603±0.162
	Maximum Respiration (R_{max})	0.574±0.037	0.529±0.161
	Resting Metabolic Rate ($MR_{resting}$)	0.577±0.038	0.524±0.169

Supplementary Table S5. The size structured prey densities differed between the period of natural flow (romanised) and modified low flow (italicised) (mean ± SD shown).

Parameter	Stretch						
	1	2	3	4	5	6	7
Drift Density (invertebrate .m^3)							
Aquatic							
1-3mm	2.1±1.4	2.1±1.4	2.9±2.0	1.8±1.2	0.2±0.1	1.4±0.9	3.1±2.1
	3.6±0.6	*3.6±0.6*	*5.1±0.9*	*3.1±0.5*	*0.4±0.1*	*2.4±0.4*	*5.5±0.9*
3-5mm	1.6±0.3	1.6±0.3	2.3±0.5	1.4±0.3	0.2±0.0	1.1±2.2	2.4±0.5
	1.6±0.3	*1.6±0.3*	*2.2±0.4*	*1.4±0.2*	*0.2±0.0*	*1.0±0.2*	*2.4±0.4*
5-7mm	1.8±9.7	1.8±9.7	2.5±1.0	1.5±0.6	0.2±0.1	1.2±0.5	2.7±1.1
	1.2±0.2	*1.2±0.2*	*1.7±0.3*	*1.1±0.2*	*0.1±0.0*	*0.8±0.1*	*1.9±0.3*
7-9mm	1.0±0.8	1.0±0.8	1.4±1.1	0.9±0.7	0.1±0.1	0.1±0.0	1.5±1.2
	0.1±0.0	*0.1±0.0*	*0.1±0.0*	*0.1±0.0*	*0.0±0.0*	*0.7±0.5*	*0.1±0.0*
9-12mm	0.1±0.0	0.1±0.0	0.2±0.0	0.1±0.0	0.0±0.0	0.1±0.0	0.2±0.1
	0.2±0.0	*0.2±0.0*	*0.3±0.1*	*0.2±0.0*	*0.0±0.0*	*0.2±0.0*	*0.4±0.1*
Terrestrial							
1-3mm	0.3±0.2	0.3±0.2	0.3±0.3	0.1±0.1	0.0±0.0	0.1±0.1	3.9±3.5
	0.4±0.4	*0.4±0.4*	*0.4±0.3*	*0.2±0.2*	*0.0±0.0*	*0.1±0.1*	*5.3±5.3*
3-5mm	0.1±0.1	0.1±0.1	0.1±0.1	0.1±0.1	0.0±0.0	0.0±0.0	1.9±1.7
	0.0±0.0	*0.0±0.0*	*0.0±0.0*	*0.0±0.0*	*0.0±0.0*	*0.0±0.0*	*0.3±0.4*
5-7mm	0.0±0.0	0.0±0.0	0.0±0.0	0.0±0.0	0.0±0.0	0.0±0.0	1.1±1.4
	0.0±0.0	*0.0±0.0*	*0.0±0.0*	*0.0±0.0*	*0.0±0.0*	*0.0±0.0*	*0.4±0.5*
7-9mm	0.0±0.0	0.0±0.0	0.0±0.0	0.0±0.0	0.0±0.0	0.0±0.0	0.1±0.1
	0.0±0.0	*0.0±0.0*	*0.0±0.0*	*0.0±0.0*	*0.0±0.0*	*0.0±0.0*	*0.0±0.0*
9-12mm	0.0±0.0	0.0±0.0	0.0±0.0	0.0±0.0	0.0±0.0	0.0±0.0	0.1±0.1
	0.0±0.0	*0.0±0.0*	*0.0±0.0*	*0.0±0.0*	*0.0±0.0*	*0.0±0.0*	*0.0±0.0*

Supplementary Table S6. The published submodels and field study specific parameters used within FishMORPH to create virtual fish with similar bioenergetics and feeding behaviours to young-of-the-year (YOY) and one year old (1+) Atlantic salmon (*Salmo salar*) and brown trout (*Salmo trutta*).

Forager Parameters	Unit	Value / formula	Source
Salmonid energy density (E_f)	$kJ.g.ww^{-1}$	5.93	[63]
Mean fork length (FL)<br>- YOY fish<br>- 1+ fish	mm	 96<br>186	This study
Fork length (mm) : weight (g)		$44.688 \times W^{0.2681}$	This study
Territory Size<br>- YOY fish<br>- 1+ fish	m^2	$\log_{10} TS = 2.61 \times FL_{cm} - 2.83$<br>0.64<br>3.04	[21], this study
Bioenergetic equations	$cal.day^{-1}$		[29,64]
- Standard (R_s) and Maximum (R_m) Metabolic Rate		$a \times W^{b_1} \times e^{b_2 \times T}$	
- Digestion metabolic rate (R_d)		$R_{max} - R_s$	
- Feeding metabolic rate (MR_f)		$R_s + R_d + SC_{feeding}$	
- Resting Metabolic Rate (MR_f)		$R_s + R_d + SC_{resting}$	
- Energy loss through faeces (F) and urea (U)		$F + U = 0.31 \times C_{max}$	
- Max Consumption Rate (C_{max})		$a \times W^{b_1} \times e^{b_2 \times T}$	
Swimming bioenergetic cost (SC)	$cal.day^{-1}$	$a \times W^{b_1} \times e^{b_2 \times T} \times^{b_3 \times V}$	[61,68,69]

Handling Time (*HT*)	hour	$3600\times\frac{1}{2}\times RD\times\left(\frac{1}{V_{\max}}+\frac{1}{Patch_{Velocity}}\right)$	[61,70]
Maximum Speed Velocity (V_{max})	$m.s^{-1}$	$36.23\times FL^{0.19}$	[61]
Capture Probability Success (CPS)	%	$104.8\times\left(151.9\times Patch_{Velocity}\right)$	[62]
Capture Area (CA)	m^2	$\begin{cases} 2\times RD\times RD & \text{if } Patch_{Depth}\geq RD \\ 2\times RD\times Patch_{Depth} & \text{if } Patch_{Depth}<RD \end{cases}$	[27]
Reaction Distance (RD)	m	$\begin{cases} 12\times PL_{mean}\times\left(1-e^{FL_{mm}}\right) & \text{if } Daylight=1 \\ 0 & \text{if } Daylight=0 \end{cases}$	[71], this study
Rate of encounter for each prey category (*i*) (RE)	$invertebrate.hr^{-1}$	$ER_f=3600\times DD_f\times V_{p,t}\times CA_f$	This study
Capture rate for each prey category (*i*) (CR)	$\underline{invertebrate.hr}^{-1}$	$CR_{i,t}=CPS\times ER\Big/\left(1+\sum_{i}^{10}CPS\times ER_{i,t}\times HT\right)$	[70]
Prey length (PL)<br>- minimum<br>- maximum	mm	$PL_{\min}=0.115\times FL_{mm}$<br>$PL_{\max}=0.452\times FL_{mm}$	[61,72]

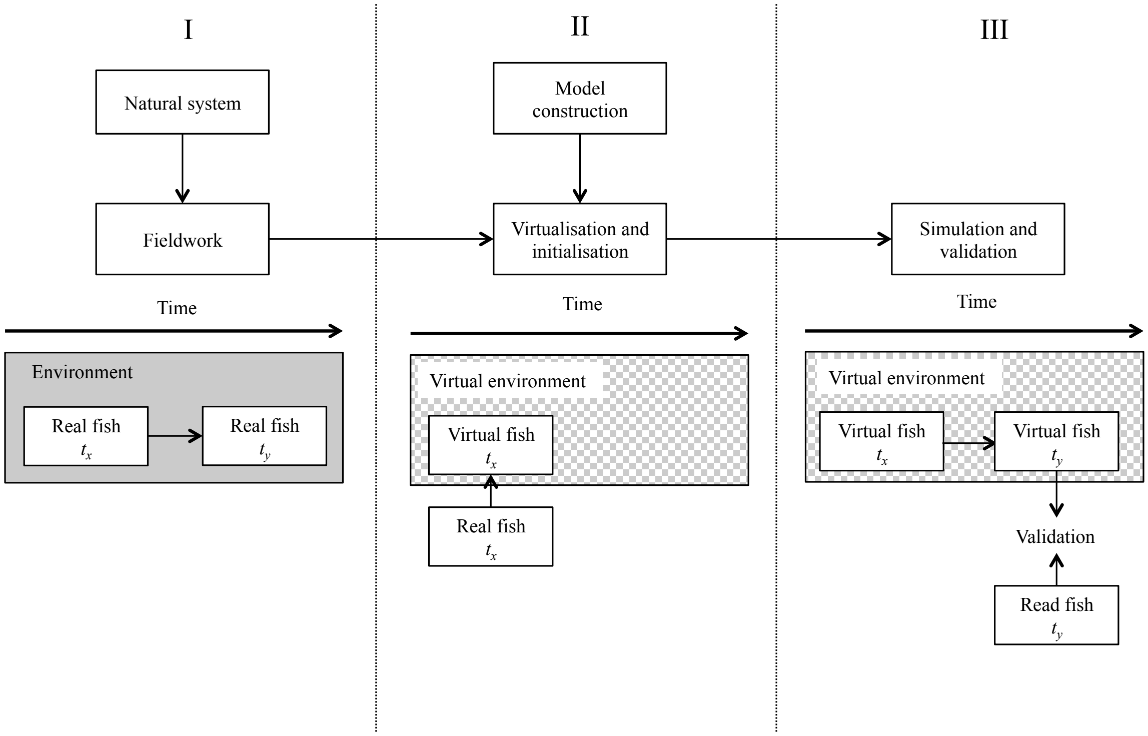

Supplementary Figure S1

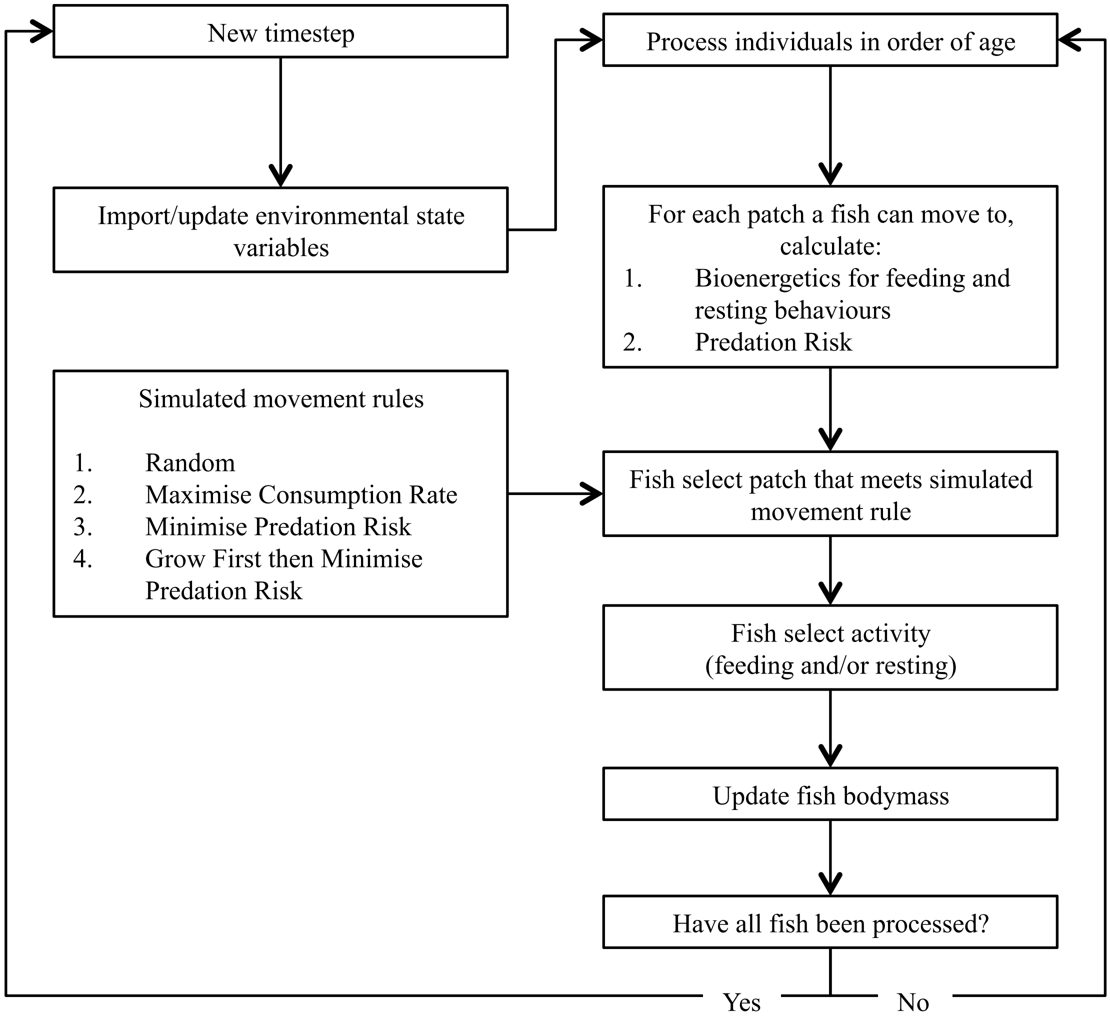

Supplementary Figure S2

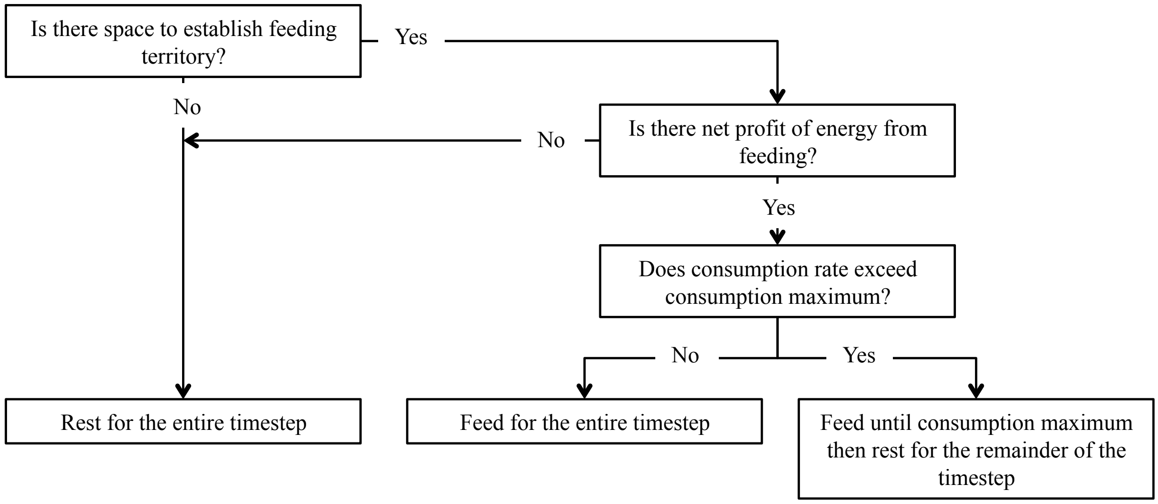

Supplementary Figure S3
