# Supplementary figures and images for: FishMORPH - An agent-based model to predict salmonid growth and distribution responses under natural and low flows
Source: Sci Rep. 2016 Jul 19;6:29414. doi: 10.1038/srep29414 (PMC4949470; doi:10.1038/srep29414)

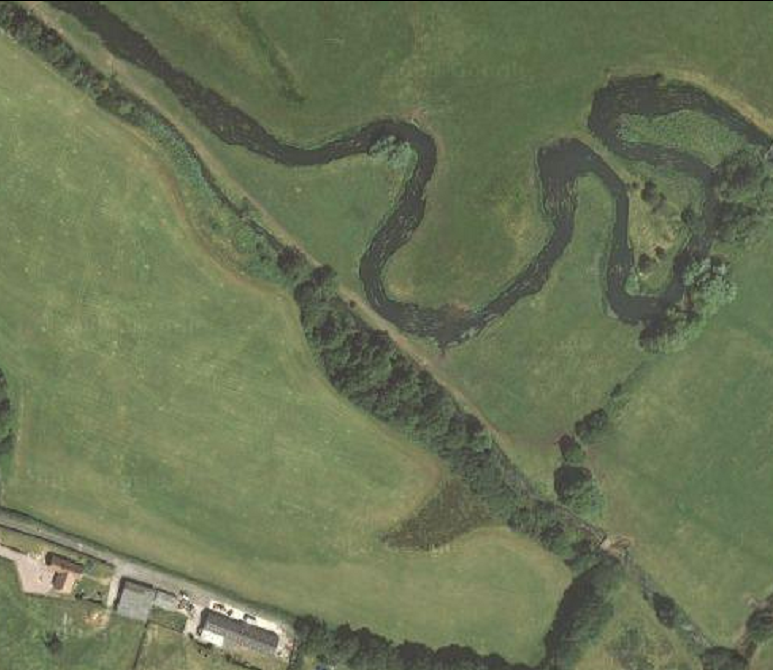

Supplement: Supplementary Information 2 [file srep29414-s2.zip › SR_FishMORPH_Files/Parameters/Background.bmp]
