# Supplementary material for: FishMORPH - An agent-based model to predict salmonid growth and distribution responses under natural and low flows
Source: Sci Rep. 2016 Jul 19;6:29414. doi: 10.1038/srep29414 (PMC4949470; doi:10.1038/srep29414)
Supplement: Supplementary Information 2 [file srep29414-s2.zip › SR_FishMORPH_Files/Morph User Guide.doc]

**MORPH - An individual-based model to predict the effect of environmental change on animal populations**

**User Guide**

Richard A. Stillman *

Centre for Ecology and Hydrology, Winfrith Technology Centre, Winfrith Newburgh, Dorchester, Dorset DT2 8ZD, UK.

* Present address. School of Conservation Sciences, Bournemouth University, Talbot Campus, Poole, Dorset, BH12 5BB, UK. Email. rstillman@bournemouth.ac.uk.

**Contents**

1. Overview	1

2. Installing	2

3. Running	3

4. User interface	4

4. Structure of parameter file	8

6. Description of parameter file	10

7. Example parameter file	54

7. Structure of result files	63

8. Description of results files	64

# 1. Overview

This is a user guide for MORPH, an individual-based model designed to predict how animal populations are affected by environmental change (e.g. habitat loss, changes in human disturbance, climate change, mitigation measures in compensation for developments and changes in population size itself). MORPH considers populations of one or more forager types (e.g. species, age classes or sexes within a population), within an environment comprised of a number of patches (e.g. habitat types), differing in their location, environmental characteristics and food resources. Foragers consume resources (e.g. vegetation, prey species) from these patches, from which they digest the resource components they require to survive (e.g. energy, nutrients). MORPH predicts how individual foragers respond to environmental change by altering their feeding location (patch), consuming different food (resources) or adjusting the amount of time spent feeding. The central assumption of MORPH is that foragers behave in ways that maximise their chances of survival. MORPH has been designed to be very flexible, so that it can produce both general predictions (when parameterised in a very simple way), and predictions for specific systems (when parameterised using detailed system-specific data). The purpose of this guide is to describe how to install, parameterise and run MORPH. A description of MORPH and examples of its use are given in.

*Stillman, R. A. (submitted to Ecological Modelling). MORPH - An individual-based model to predict the effect of environmental change on animal populations.*

# 2. Installing

MORPH is written in Borland Delphi 7 (www.borland.com), and runs in Microsoft Windows 98 or later. MORPH is installed by copying the following files into an empty folder (e.g. C:\MORPH).

**Type	File name	Description**

Compiled files	MORPH.EXE	Executable model file

UCALC32.DLL	Commercially available maths parser to	evaluate equations

Source files	MORPH.DPR	Delphi project file

MAINFORM.PAS	Unit to display main window and control	other units

STARTFORM.PAS	Unit to display startup window

PARSERUNIT.PAS	Unit to control interaction with maths	parser

GLOUNIT.PAS	Unit to control global model environment

PATUNIT.PAS	Unit to control model patches

FORUNIT.PAS	Unit to control model foragers

UCALC.PAS	Maths parser unit to interface with Delphi

Other Delphi files	MAINFORM.DFM	Stores details of main window

STARTFORM.DFM	Stores details of startup window

MORPH.RES	Stores details of model

Parameter files	EXAMPLE.PAR	Parameter file

BACKGROUND.BMP	Image drawn behind patches on graphical	display

FOREGROUND.BMP	Image drawn in front of patches on	graphical display

Windows shortcut	RUNMORPH.LNK	Shortcut to run MORPH from C:\MORPH.

The only files required to run MORPH are MORPH.EXE, UCALC32.DLL and the parameter files. MORPH.EXE is the program code for MORPH. UCALC32.DLL is a commercially available maths parser, UCALC, which enables MORPH to read in equations from the parameter file. No licence is required to use UCALC as long as MORPH is not recompiled.

The remaining files are MORPH’S source code (MORPH.DPR and *.PAS files), files required by Delphi and a Windows shortcut to run MORPH from C:\MORPH. MORPH’s source files can be viewed in any text editor, but need to be opened within Delphi in order to see the interactions between different parts of the code, to change the code or to recompile the code to generate a new executable file. A licence to use UCALC, obtainable from WWW.UCALC.COM, is required if MORPH is recompiled. Once a licence code is purchased replace “Enter licence here” with the licence code in the command “ucLicense(‘Enter licence here’);” in the PARSERUNIT.PAS. MORPH will not run correctly until a valid UCALC licence has been supplied.

# 3. Running

To run MORPH, MORPH.EXE and UCALC32.DLL need to be placed in the same folder, but parameter files can be placed in any folder. MORPH is best run in one of two ways: (1) from a Windows shortcut; or (2) from a batch program capable of sequentially running multiple commands from a batch file. A shortcut is the best way to run a single parameterisation of MORPH (e.g. when showing a demonstration), and the batch option is the best way of running multiple parameterisations (e.g. when running a set of simulations each based on a different set of parameters). Whether in a shortcut or a batch file, the command line required to run MORPH has the following structure (optional elements are enclosed within {}).

{PATH}MORPH.EXE {PARPATH}PARFILENAME RESPATH RESFILEEXT {/RUNMODE}

where {PATH} = optional path to folder holding MORPH.EXE, {PARPATH} = optional path to folder holding parameter file, PARFILENAME = name of parameter file, RESPATH = path to folder in which results will be saved, RESFILEEXT = extension of result files and {/RUNMODE} = option to set MORPH’S run mode. For example, to run the example parameter file, place the following command in a Windows shortcut (or use the file RUNMORPH.LNK, which is a shortcut set up to run this command).

C:\MORPH\MORPH.EXE C:\MORPH\EXAMPLE.PAR C:\MORPH TXT

Then ensure that MORPH.EXE, UCALC32.DLL, EXAMPLE.PAR, BACKGROUND.BMP AND FOREGROUND.BMP are in the folder C:\MORPH. MORPH can then be run be running the shortcut. This will cause MORPH to read in parameters from EXAMPLE.PAR, and write results to C:\MORPH with the file extension TXT.

As MORPH runs it writes results to the following six text files, with file extensions determined by the RESFILEEXT command line parameter, in a folder determined by RESPATH command line parameter.

LOG – General summary of the simulation and the parameter file.

GLOBAL – Global results.

PATCH – Patch-specific results.

RESOURCE – Resource-specific results.

DIET – Diet-specific results.

FORAGER – Forager-specific results.

Three run modes are possible: (1) “/MAX” runs MORPH in a maximised window; (2) “/MIN” runs MORPH in a minimised window; (3) “/CHECK” simply reads and checks the parameter file. The default mode is /MAX which is used if no mode is set on the command line. MORPH runs more quickly in the /MIN mode because no time is spent updating the screen. The run mode sets the window state at the start of a simulation but this can be changed while MORPH is running.

# 4. User interface

Unless MORPH is run in /MIN mode, a start up window (see example below) is shown as MORPH reads and checks the parameter file.

The window’s title bar indicates the simulation name and that parameters are being read. The text in the window shows the parameter file name and path, and what section of the parameter file is currently being read. A progress bar also shows the line within the parameter file that is currently being read. MORPH can be quit at any stage by clicking the *Quit* button. No start up window is displayed if MORPH is run in /MIN mode. An error message is displayed if any errors are found while reading the parameter file. If MORPH is run in /CHECK mode, it quits after all parameters have been read and checked.

Unless MORPH is in /MIN or /CHECK mode, the main window is displayed after all parameters have been read (see example below).

The window’s title bar indicates the simulation name and the percentage of time steps which have been completed.

**Map image (1)**

The left of the screen (1) is occupied by the Map Image which displays the location and shape of patches, and the location of foragers on or between the patches. Foragers have a black border when on a patch and a white border when moving between patches. An individual forager can be highlighted (doubled in size) by double-clicking on it in the map image, the highlight can be removed by another double-click.

**Control buttons (2)**

The bottom left of the screen contains six buttons to control the simulation (2).

- *Quit* – stops the simulation.
- *Pause* – pauses the simulation. When the simulation is paused this button caption changes to *Resume* and is used to restart the simulation.
- *Hide* –hides all screen output and so increases the speed at which the simulation runs. When graphics are hidden the button caption changes to *Display* and is used to redisplay screen output.
- *Faster* and *Slower* – used to alter the time delay between displaying the results of consecutive time steps.
- *Next* – can be used to advance the simulation one time step at a time when the simulation is paused.

**Information area (3)**

The right of the screen contains five tab-sheet pages which display information about the current simulation. The *Details* tab-sheet is displayed when MORPH starts running and lists all model results during each time step. The five tables on this tab-sheet (from top to bottom) display global results applying throughout a simulation, patch-specific results, resource-specific results, diet-specific results and forager-specific results. Each of the columns can be sorted by double-clicking on the column name. An individual forager can be highlighted by double-clicking its number (left hand column in bottom table), and un-highlighted by another double-click. The highlighted forager is drawn larger on the map image and its row highlighted in the forager-specific table.

The *Variables* tab-sheet (see example below) displays global and patch-specific variables (these elements are explained later in the guide).

The *Resources* tab-sheet (see example below) displays the distribution of resources and resource components between different patches (these elements are explained later in the guide). The top panel shows the display colour of each resource, and the bottom panel shows the density of each component within each resource on each patch. The units of density depend on the units of component density within resources and the units of resource density within patches (e.g. if component density is measured in g per unit of resource and resource density is measured m^-2^, the values are g of component m^-2^).

The *Foragers* tab-sheet (see example below) shows the fates and distribution of foragers (these elements are explained later in the guide). The top panel shows the display colour of each forager type. The middle panel shows the number of individuals that are still immigrating to the system, the number present in the system, the number that have emigrated from the system and the number that have died from each source of mortality. The bottom panel shows the number of individuals on each patch and the number that are moving between patches.

The *Parameters* tab-sheet (see example below) shows a general summary of the simulation and lists the parameter file.

# 4. Structure of parameter file

MORPH reads parameters from a single text file. It does not assume any particular units (e.g. for time, distance or area), but the user must ensure that consistent units are used throughout the parameter file (e.g. that time is always measured in s, and that distance is always measured in m).

The parameter file is divided into four sections with the following titles.

******************** **GENERAL PARAMETERS** ********************

******************** **GLOBAL PARAMETERS** ********************

******************** **PATCH PARAMETERS** ********************

******************** **FORAGER PARAMETERS** ********************

Each section is further divided into individual parameters. Each parameter entry has the following components.

**Parameter description**

A single line of text describing the parameter, the type of data expected and any constraints on parameter values. The line starts with a description of the parameter, followed by the parameters type and constraints contained within brackets.

**Parameter value(s)**

Single or multiple lines holding the parameter value(s). Single line parameters consist of a single line containing parameter values. Multiple line parameters are used to read parameters for several model entities (e.g. each patch, patch variable or forager type). Each line of a multiple line parameter starts with a code to indicate the model entity for which a value is being read, followed by the parameter value (e.g. P1V2 100 sets the value of patch variable 2 on patch 1 to 100). Model entities must be defined in order (e.g. P1V1, P1V2, P2V1, P2V2 etc.).

**Types of parameter**

Seven types of parameters can be read.

*Name* – The name of the simulation itself or a model entity. With the exception of the name of the simulation itself, names must be unique, and can only contain letters and numbers, and cannot start with a number. An error message is displayed if the parameter file contains a duplicate name or if a name contains non-alphanumeric characters or starts with a number.

*File* – A filename. An error message is displayed if the file does not exist. The file name can include either an absolute path or path relative to the current active folder.

*Coordinates* – A pair of X and Y coordinates separated by a space character.

*Colour* – Colour code. Colour codes can be specified using the rgb function (see below).

*Number* – A number. The range of valid numbers may be limited for some parameters. MORPH recognises “.” as a decimal separator in numbers. Care should be taken to ensure that this convention is adhered to.

*Formula* – An equation, which may incorporate other parameter values that are automatically defined by MORPH or have been defined previously in the parameter file. Formulae can either be single numbers (e.g. 10), functions of numbers (e.g. 10 * 10) or functions containing both numbers and previously read parameters (e.g. 10 * *PatSize*, where *PatSize* = size of a patch).

MORPH recognises “,” as a function argument separator. Care should be taken to ensure that this convention is adhered to.

MORPH checks that formulae are valid before they are executed, but does not check for errors that may occur when a formula is executed (e.g. division by zero or the log of zero). If such an error occurs, MORPH will display an error message and stop running. Care should, therefore, be taken in choice of formulae to ensure that such errors do not occur.

Formulae are evaluated using the UCalc maths parser (WWW.UCALC.COM), so any functions valid in UCalc can also be used in MORPH. The following non-standard UCalc functions are also available.

uniform(min, max)	Generates a random number drawn from a uniform distribution within given range (*min* to *max*)

normal(mean, sd, min, max)	Generates a random number within the given range (*min* to *max*), drawn from a normal distribution with given mean (*mean*) and standard deviation (*sd*)

if(condition, true, false)	Can be used in addition to the standard UCalc iff function. Executes statement *true* if *condition* is true, and executes statement *false* if *condition* is false.

rgb(red,green,blue)	Generates a colour code (see above) with a colour specified by the values of *red*, *green* and *blue*. Each of these parameters is a value in the range 0-255, specifying the amount of red, green and blue in the colour (e.g. rgb(255,128,0) returns a colour code for orange).

*Variable file* – A list of parameter values stored in a variable file, containing one parameter value for each model time step. Some parameters can be defined either using a formula or a variable file. The variable file is identified in a parameter file by preceding its name (including path) by the “@” character. Variable files have the following structure.

Example variable file

T1 100

T2 50

T3 150

T4 40

T5 60

T6 170

T7 100

T8 10

T9 150

T10 90

The first line describes the contents of the variable file. Subsequent lines contain a time step code (e.g. T1), followed by a space character, followed by the value for a time step (e.g. 100). The number of time steps in a variable file must equal or exceed the number of time steps in a simulation.

# 6. Description of parameter file

This section describes each of MORPH’S parameters, explaining the type of data that each parameter can accept, and in the case of formula parameters, which previously defined parameters can be included in formulas. The section also indicates (boxed text) the position in the parameter file at which different parameters are defined, and are therefore available for subsequent formulae. The sections are listed in the same order as in the parameter file as the sequence in which parameters are defined determines which are available for use in formulae.

Each parameter description contains five elements, as follows:

1. Parameter number, name and description
2. Parameter type – the type of data expected for the parameter.
3. Potential parameters – the set of previously defined parameters that can be used by the parameter
4. Header – the text used to describe the parameter in the parameter file
5. Examples – a list of examples (in *italics* and “quotes”) with explanations (after a “–” character)

********************** General parameters **********************

**Parameter 1 – Simulation name**

Name of the simulation. This appears on the title bar at the top of MORPH’s main window, and on the Windows task bar.

**Parameter type**

Name

**Potential parameters**

None

**Header**

*“Name of simulation (text):”*

**Examples**

*“Shorebird model”* – Simulation name set to “Shorebird model”

*“Wildfowl model”* – Simulation name set to “Wildfowl model”

**Parameter 2 – Background image file**

Name of file containing a bitmap which will be drawn on the map image behind patches and foragers, or a blank line if no background image is required. A background bitmap can be used to add detail to the map image. The bitmap is scaled to fit exactly within the map image. Therefore, the width and height of the foreground image should have the same proportions as the width and height of the map image (set with the minimum (parameter 4) and maximum (parameter 5) bounding coordinates) to prevent the background image being distorted when displayed.

**Parameter type**

File

**Potential parameters**

None

**Header**

*“Name of map background image file (file name or blank line):”*

**Examples**

“*BACKIMAGE.BMP*”

– Background image drawn from file “BACKIMAGE.BMP”

“*C:\IMAGES\BACKIMAGE.BMP*”

– Background image drawn from file “C:\IMAGES\BACKIMAGE.BMP”

**Parameter 3 – Foreground image file**

Name of file containing a bitmap which will be drawn on the map image in front of patches and foragers, or a blank line if no foreground image is required. A foreground bitmap can be used to add detail to the map image. The bitmap is scaled to fit exactly within the map image. Therefore, the width and height of the foreground image should have the same proportions as the width and height of the map image (set with the minimum (parameter 4) and maximum (parameter 5) bounding coordinates) to prevent the background image being distorted when displayed. Part of the foreground image must be transparent in order to view the background image, patches and foragers. To achieve this, all parts of the foreground image with the same colour as the bottom left pixel of the image are displayed as transparent.

**Parameter type**

File

**Potential parameters**

None

**Header**

*“Name of map foreground image file (file name or blank line):”*

**Examples**

“*FOREIMAGE.BMP*”

– Foreground image drawn from file “FOREIMAGE.BMP”

“*C:\IMAGES\FOREIMAGE.BMP*”

– Foreground image drawn from file “C:\IMAGES\FOREIMAGE.BMP”

**Parameter 4 – Map image minimum coordinates**

X and Y coordinates representing bottom left corner of map image. Any units can be used, but these must be consistent with the units used for map boundaries (parameter 22).

**Parameter type**

Coordinates

**Potential parameters**

None

**Header**

*“Minimum coordinates of map image (coordinates):”*

**Examples**

“*0 0*” – Minimum map image coordinates set to X = 0 and Y = 0

*“-100 -1000*” – Minimum map image coordinates set to X = -100 and Y = -1000

**Parameter 5 – Map image maximum coordinates**

X and Y coordinates representing top right corner of map image. Any units can be used, but these must be consistent with the units used for map boundaries (parameter 22).

**Parameter type**

Coordinates

**Potential parameters**

None

**Header**

*“Maximum coordinates of map image (coordinates):”*

**Examples**

“*100 1000*” – Maximum map image coordinates set to X = 100 and Y = 1000

“*-10 0*” – Maximum map image coordinates set to X = -10 and Y = 0

**Parameter 6 –Maths parser memory size**

Relative amount of memory used by maths parser to store parameters and expressions. The default setting is 1, and should be sufficient for all except the most complicated models. However, this can be increased if an error message indicates that the parameter and expression memory is exceeded. The value should be increased by small amounts (e.g. 1.1, 1.2, 1.3), until the error message is not generated, to determine the appropriate size of parser memory.

**Parameter type**

Number

**Potential parameters**

None

**Header**

*“Relative size of parameter and expression memory (>=1):”*

**Examples**

“*1*” – Parser memory size set to default size

“*2*” – Parser memory size set to double default size

********************** Global parameters **********************

**Parameter 7 – Number of time steps**

Number of time steps in simulation. A simulation must have at least one time step.

**Parameter type**

Number (>= 1)

**Potential parameters**

None

**Header**

*“Number of time steps (>=1):”*

**Examples**

“*100*” – The number of time steps is set to 100

“*1000*” – The number of time steps is set to 1000

**Parameter 8 – Duration of time steps**

The duration of each time step. Any unit of time can be used, but the same unit of time must be used throughout the parameter file (e.g. if the unit of time is s, all subsequent parameters that refer to time must also be measured in units of s).

**Parameter type**

Number (> 0)

**Potential parameters**

None

**Header**

*“Duration of each time step (>0):”*

**Examples**

“*1*” – The duration of each time step is set to one unit of time

“*1.5*” – The duration of each time step is set to one and a half units of time

**Parameters defined**

**TimeStep**	Current time step number (e.g. = 50 when the simulation has reached time step 50)

**NumTimeStep**	Total number of time steps in simulation

**TimeStepLength**	Duration of each time step (measured in time step units)

**Parameter 9 – Number of global variables**

Number of global variables. Global variables apply throughout the simulated environment (e.g. day number, time of day, temperature).

**Parameter type**

Number (>= 0)

**Potential parameters**

None

**Header**

*“Number of global variables (>=0):”*

**Examples**

“2”

– The number of global variables is set to 2. 2 global variables must be defined in subsequent parameters.

“5”

– The number of global variables is set to 5. 5 global variables must be defined in subsequent parameters.

**Parameter 10 –Global variable names**

Name of each global variable. Global variables cannot contain spaces or non-alphanumeric characters, and cannot start with a number.

**Parameter type**

Name for each global variable. The global variable is indicted by “V” followed by the global variable number.

**Potential parameters**

None

**Header**

*“Name of each global variable (text without spaces):”*

**Examples**

“*V1 Day*” – Global variable 1 is named “Day”

“*V2 Time*” – Global variable 2 is named “Time”

“*V3 Temperature*” – Global variable 3 is named “Temperature”

**Parameters defined**

**Global variables**	Value of each global variable during each time step. One parameter is defined for each global variable, with the same name as the global variable (e.g. Day, Time, Temperature).

**Parameter 11 – Global variable values**

The value of each global variable during each time step is either calculated from a formula or read from a text file. The formula can include any of the previously defined parameters including previous global variables (e.g. the second global variable can depend on the values of the first global variable) or it can be a fixed value. Values are read from a file if the “@” symbol precedes the name of an existing file (e.g. @TEMPERATURE.TXT).

**Parameter type**

Formula or variable file for each global variable. The global variable is indicted by “V” followed by the global variable number.

**Potential parameters**

Time step, Number of time steps, Time step length. Previously defined global variables.

**Header**

*“Value of each global variable (formula or file):”*

**Examples**

“*V1 (TimeStepLength*(TimeStep-1)) div 24*” – Calculates the current day number

“*V2 (TimeStepLength*(TimeStep-1)) mod 24*” – Calculates the current time of day

“*V3 @TEMPERATURE.TXT*”

– Reads temperature for each time step from variable file “TEMPERATURE.TXT”

**Parameter 12**

Colour in which global variable is displayed in the Variables Tab-Sheet. The colour is calculated using the rgb formula.

**Parameter type**

Colour for each global variable. The global variable is indicted by “V” followed by the global variable number.

**Potential parameters**

None

**Header**

*“Colour of each global variable (colour code):”*

**Examples**

“*V1 RGB(255,0,0)*” – Global variable 1 is displayed in red

“*V2 RGB(0,255,0)*” – Global variable 2 is displayed in green

“*V3 RGB(0,0,255)*” – Global variable 2 is displayed in blue

**Parameter 13 – Rule for whether global results are saved**

Formula to calculate whether global results are saved at the end of a time step. The formula must return one during time steps in which results are to be saved and zero during time steps in which results are not to be saved. Any previously defined parameters can be used in this calculation (e.g. TimeStep=50 saves global results during time step 50).

**Parameter type**

Number

**Potential parameters**

Time step, Number of time steps, Time step length. Global variables.

**Header**

*“Rule for whether global results are saved (1=yes; 0=no):”*

**Examples**

“*if(Day=100,1,0)*”

– Global results are only displayed on day 100. Day must have been defined previously.

“*if(Time=12,1,0)*”

– Global results are only displayed when the time = 12. Time must have been defined previously.

********************** Patch parameters **********************

**Parameter 14 – Number of patches**

Number of patches. The number of patches is fixed throughout a simulation.

**Parameter type**

Number (>= 1)

**Potential parameters**

None

**Header**

*“Number of patches (>=1):”*

**Examples**

“*5*” – The number of patches is set to 5.

“*10*” – The number of patches is set to 10

**Parameter 15 – Patch names**

Name of each patch. Patch names must be unique, cannot contain spaces or non-alphanumerical characters, and cannot start with a number.

**Parameter type**

Name for each patch. The patch is indicted by “P” followed by the patch number.

**Potential parameters**

None

**Header**

*“Name of each patch (text without spaces):”*

**Examples**

“*P1 CockleBed*” – Patch 1 is named “CockleBed”

“*P2 MusselBed*” – Patch 2 is named “MusselBed”

**Parameter 16 – Coordinate system**

Coordinate system used to represent the spatial distribution of patches. 1 = patches located on a two dimensional plane, with X and Y coordinates. 2 = patches located on the globe, with coordinates expressed as latitude and longitude and the distance between patches calculated in m. Latitude is measured as degrees north of the equator, with negative values measuring degrees south. Longitude is measure as degrees east of the prime meridian, with negative values measuring degrees west. When a two dimensional plane is used, the units of the X and Y can be in any distance measure, but the same distance measure must be used throughout the parameter file.

**Parameter type**

Number

**Potential parameters**

None

**Header**

*“Coordinate system (1=X/Y; 2=latitude/longitude):”*

**Examples**

“*1*” – The coordinate system is set to X/Y

“*2*” – The coordinate system is set to latitude/longitude with distances measured in m

**Parameter 17 – Patch sizes**

Size of each patch. Size can be expressed in either two or three dimensions, but the dimension must be consistently used throughout the parameter file. The dimensions of the patch must be measured in the same units as the coordinate system. Patches are displayed on the screen in two dimensions, even if they are three dimensional.

**Parameter type**

Number for each patch. The patch is indicted by “P” followed by the patch number.

**Potential parameters**

None

**Header**

*“Size of each patch (>0):”*

**Examples**

“*P1 100*” – The size of patch 1 is set to 100 units

“*P2 10000*” – The size of patch 2 is set to 10000 units

**Parameter 18 – Patch central coordinates**

The central coordinates of each patch expressed in the appropriate coordinate system.

**Parameter type**

Coordinates for each patch. The patch is indicted by “P” followed by the patch number.

**Potential parameters**

None

**Header**

*“Central coordinates of each patch (coordinates):”*

**Examples**

“*P1 100 100*”

– The central coordinates of patch 1 are set to X = 100, Y = 100 (coordinate system = 1)

“*P2 3 -4*”

– The central coordinates of patch 2 are set to latitude = 3^o^, longitude = -4^o^ (coordinate system = 2)

**Parameters defined**

**PatchNumber**	Number of each patch

**PatchSize**	Size of each patch

**PatchX, PatchY**	Patch X and Y coordinates (only defined if coordinate system = 1)

**PatchLat, PatchLong**	Patch latitude and longitude (only defined if coordinate system = 2)

**Parameter 19 – Number of map boundaries**

The number of map boundaries used to graphically represent each patch in the map image. Each map boundary is drawn as a polygon on the map image. Within MORPH each patch is considered to be continuous, but patches may be displayed as one or more polygons.

**Parameter type**

Number (>= 1) for each patch. The patch is indicted by “P” followed by the patch number.

**Potential parameters**

None

**Header**

*“Number of map boundaries displayed for each patch (>=1):”*

**Examples**

“*P1 1*” – Patch 1 is displayed by one map boundary

“*P2 2*” – Patch 2 is displayed by two map boundaries

**Parameter 20 – Number of map boundary coordinates**

The number of vertices for each of the polygons representing each patch. At least 3 vertices are required to represent a polygon. The polygon does not need to be closed (i.e. the last vertex does not need to have the same coordinates as the first).

**Parameter type**

Number (>= 3) for each patch and boundary. The patch is indicted by “P” followed by the patch number, and the boundary by “B” followed by the boundary number.

**Potential parameters**

None

**Header**

*“Number of coordinates for each map boundary of each patch (>=3):”*

**Examples**

“*P1B1 4*” – Boundary 1 of patch 1 has 4 vertices

“*P2B14*” – Boundary 1 of patch 2 has 4 vertices

“*P2B2 3*” – Boundary 2 of patch 2 has 3 vertices

**Parameter 21 - Map boundary coordinates**

The coordinates of each vertex for each of the polygons representing each patch. These coordinates must be in the same units as those used to define the minimum and maximum bounding coordinates of the map image. The polygon does not need to be closed (i.e. the coordinates of the first and last vertices do not need to be the same).

**Parameter type**

Coordinates for each patch, boundary and vertex location. The patch is indicted by “P” followed by the patch number, the boundary by “B” followed by the boundary number and the vertex location by “L” followed by the vertex number.

**Potential parameters**

None

**Header**

*“Coordinates for each map boundary of each patch (coordinates):”*

**Examples**

“*P1B1L1 50 50*” – Boundary 1 of patch 1 is displayed as a square

“*P1B1L2 150 50*”

“*P1B1L3 150 150*”

“*P1B1L4 50 150*”

“*P2B1L1 50 200*” – Boundary 1 of patch 2 is displayed as a square

“*P2B1L2 150 200*”

“*P2B1L3 150 300*”

“*P2B1L4 50 300*”

“*P2B2L1 50 400*” – Boundary 2 of patch 2 is displayed as a triangle

“*P2B2L2 100 500*”

“*P2B2L3 150 400*”

**Parameter 22 – Number of patch variables**

Number of patch variables. Patch variables apply to individual patches.

**Parameter type**

Number (>= 0)

**Potential parameters**

None

**Header**

*“Number of patch variables (>=0):”*

**Examples**

“*0*” – The number os patch variables is set to 0

“*2*” – The number os patch variables is set to 2

**Parameter 23 – Patch variable names**

Name of each patch variable. Patch variable names must be unique, cannot contain spaces or non-alphanumerical characters, and cannot start with a number.

**Parameter type**

Name for each patch variable. The patch variable is indicted by “V” followed by the patch variable number.

**Potential parameters**

None

**Header**

*“Name of each patch variable (text without spaces):”*

**Examples**

“*V1 Available*” – Patch variable 1 is named “Available”

“*V2 WaterDepth*” – Patch variable 2 is named “Water depth”

**Parameter 24 – Patch variable colours**

Colour in which patch variable is displayed in the Variables Tab-Sheet. The colour is calculated using the rgb formula.

**Parameter type**

Colour for each patch variable. The patch variable is indicted by “V” followed by the patch variable number.

**Potential parameters**

None

**Header**

*“Colour of each patch variable (colour code):”*

**Examples**

“*V1 RGB(255,0,0)*” – Patch variable 1 is displayed in red

“*V2 RGB(0,255,0)*” – Patch variable 2 is displayed in green

**Parameters defined**

**Patch variables**	Value of each patch variable during each time step. This parameter has the same name as the patch variable to which it refers (e.g. Available, WaterDepth).

**Parameter 25 – Patch variable values**

Value of each patch variable during each time step either calculated from a formula or read from a variable file. The formula can include any of the previously defined parameters including previous patch variables (e.g. the second patch variable can depend on the values of the first patch variable). Values are read from a file if the “@” symbol precedes the name of an existing file.

**Parameter type**

Formula or file for each patch and patch variable. The patch is indicted by “P” followed by the patch number and the patch variable by “V” followed by the patch variable number.

**Potential parameters**

Time step, Number of time steps, Time step length. Global variables. Patch number, Patch size, Patch location. Previously defined patch variables.

**Header**

*“Value of each patch variable on each patch (formula or file):”*

**Examples**

“*P1V1 if((Time > 6) and (Time < 18), 1, 0)*”

– Value is 1 between a time of 6 and 18, and otherwise 0

“*P1V2 Uniform(0,1)*” – Value is drawn from a uniform distribution between 0 and 1

“*P2V1 Normal(1, 0.1, 0, 2)*”

– Value is drawn from a normal distribution with mean = 1, sd = 0.l, min = 0 and max = 2

“*P2V2 @WATERDEPTH.TXT*” – Value is read from the file WATERDEPTH.TXT

**Parameter 26 – Rule for whether patch results are displayed**

Formula to calculate whether patch results are displayed in the Variables, Resources and Foragers tab-sheets. The formula must return one for patches which are to be displayed and zero for patches which are not to be displayed. Any previously defined parameters can be used in this calculation (e.g. if(PatchNum <= 10, 1, 0) only displays results for patches 1 to 10).

**Parameter type**

Number

**Potential parameters**

Time step, Number of time steps, Time step length. Global variables. Patch number, Patch size, Patch location. Patch variables.

**Header**

*“Rule for whether patch results are displayed (1=yes; 0=no):”*

**Examples**

“*if(Day=200, 1, 0)*” – Patch results are displayed on day 200

“*if(PatchNumber>5, 1, 0)*” – Patch results are displayed for patches with numbers over 5

**Parameter 27 – Patch colours**

Colour in which patch is displayed in the Map Image and Variables, Resources and Foragers tab-sheets. The colour is calculated using the rgb formula. Any previously defined parameters can be used in this calculation (e.g. if(TimeStep<=100, rgb(255,0,0), rgb(0,255,0)) displays a patch in red for time steps 1 to 100, and in green for subsequent time steps).

**Parameter type**

Colour for each patch. The patch is indicted by “P” followed by the patch number.

**Potential parameters**

Time step, Number of time steps, Time step length. Global variables. Patch number, Patch size, Patch location. Patch variables.

**Header**

*“Colour of each patch (colour code):”*

**Examples**

“*P1 if((WaterDepth>0), rgb(0,0,255), rgb(0,255,0))*”

– Patch blue if water depth greater than zero, otherwise green

“*P2 if((Day<5), rgb(255,0,0), rgb(0,255,0))*” – Patch red until day 5, and then green

**Parameter 28 – Number of resources**

Number of resources. Resources are consumed by foragers.

**Parameter type**

Number (>= 1)

**Potential parameters**

None

**Header**

*“Number of resources (>=1):”*

**Examples**

“2” – The number of resources is set to 2

“5” – The number of resources is set to 5

**Parameter 29 – Resource names**

Name of each resource. Resource names must be unique, cannot contain spaces or non-alphanumerical characters, and cannot start with a number.

**Parameter type**

Name for each resource. The resource is indicated by “R” followed by the resource number.

**Potential parameters**

None

**Header**

*“Name of each resource (text without spaces):”*

**Examples**

“*R1 Cockles*” – Resource 1 is named “Cockles”

“*R2 Mussels*” – Resource 2 is named “Mussels”

**Parameter 30 – Resource colours**

Colour in which resource is displayed in the Resource Tab-Sheet. The colour is calculated using the rgb formula.

**Parameter type**

Colour for each resource. The resource is indicated by “R” followed by the resource number.

**Potential parameters**

None

**Header**

*“Colour of each resource (colour code):”*

**Examples**

“*R1 rgb(255,0,0)*” – Resource 1 is displayed in red

“*R2 rgb(0,255,0)*” – Resource 2 is displayed in green

**Parameter 31 – Resource initial density**

Initial density of resource on each patch at the start of a simulation. The units of density must be consistent with the unit of patch size (e.g. if patch size is measured in m^2^, density must be measured as m^-2^). A unit of resource density can be defined in any way (e.g. number of items m^-2^; mass m^-2^).

**Parameter type**

Formula for each patch and resource. The patch is indicated by “P” followed by the patch number and the resource by “R” followed by the resource number.

**Potential parameters**

None

**Header**

*“Initial density of each resource on each patch (formula):”*

**Examples**

“*P1R1 10000*” – Initial density of resource 1 on patch 1 is 10000

“*P1R2 100*” – Initial density of resource 2 on patch 1 is 100

“*P2R1 20000*” – Initial density of resource 1 on patch 2 is 20000

“*P2R2 200*” – Initial density of resource 2 on patch 2 is 200

**Parameters defined**

**Resource densities**	Density of each resource on each patch. This parameter is the name of the resource appended with “Dens” (e.g. “CocklesDens” is the density of a resource called “Cockles”).

**Parameter 32 – Resource density update expression**

A formula to calculate how resource density changes on each patch during each time step. These are changes in resource density additional to decreases due to resource consumption by foragers. The formula can depend on any previously defined parameter, including the density of any resource on the patch (e.g. “0.99 * CocklesDens” reduces the density of the resource named “Cockles” by 1% during each time step; “CocklesDens – 1” reduces the density of the resource by one unit during each time step; “CocklesDens” makes no changes to resource density). If the formula returns a resource density less than zero, this is set to zero by MORPH.

**Parameter type**

Formula for each patch and resource. The patch is indicated by “P” followed by the patch number and the resource by “R” followed by the resource number.

**Potential parameters**

Time step, Number of time steps, Time step length. Global variables. Patch number, Patch size, Patch location. Patch variables. Resource densities.

**Header**

*“Expression to update each resource density on each patch (formula):”*

**Examples**

“*P1R1 CocklesDens*” – Resource density is unchanged

“*P1R2 1.001 * MusselsDens*” – Resource density increases at a constant proportional rate

“*P2R1 CocklesDens – 1*” – Resource density decreases at a constant daily rate

“*P2R2 if(Day<100, MusselsDens, 0)*” – Resource density is reduced to 0 on day 100

**Parameter 33 – Number of resource components**

Number of resource components within resources. Resource components are assimilated by foragers when they consume resources. You can specify which resource components are present on which patches by setting the density of some resource components on a patch to zero.

**Parameter type**

Number (>= 1)

**Potential parameters**

None

**Header**

*“Number of resource components (>=1):”*

**Examples**

“*1*” – The number of resource components is set to 1

“*2*” – The number of resource components is set to 2

**Parameter 34 – Resource component names**

Name of each resource component. Resource component names must be unique, cannot contain spaces or non-alphanumerical characters, and cannot start with a number.

**Parameter type**

Name for each resource component. The resource component is indicated by “C” followed by the resource component number.

**Potential parameters**

None

**Header**

*“Name of each resource component (text without spaces):”*

**Examples**

“*C1 Energy*” – Resource component 1 is named “Energy”

“*C2 Nutrients*” – Resource component 2 is named “Nutrients”

**Parameter 35 – Resource component densities**

Formula to calculate the density of each resource component within each resource on each patch during each time step. Density is measured in terms of amount of component per unit of resource density (e.g. if resource density is measured as number of items m^-2^, component density is amount of component per item of resource; if resource density is measured as mass m^-2^, component density is amount of component per unit of resource mass). The formula can depend on any previously defined parameter, including the density of any resource on the patch. Resource component densities cannot be negative and MORPH displays an error message if the formula returns a negative value.

**Parameter type**

Formula for each patch, resource and resource component. The patch is indicated by “P” followed by the patch number, the resource by “R” followed by the resource number and the resource component by “C” followed by the resource component number.

**Potential parameters**

Time step, Number of time steps, Time step length. Global variables. Patch number, Patch size, Patch location. Patch variables. Resource densities.

**Header**

*“Density of each component in each resource on each patch (formula):”*

**Examples**

“*P1R1C1 100*” – Component density set to 100 throughout simulation

“*P1R1C2 10 – 0.01 * Day*” – Component density decreases at a constant daily rate

“*P1R2C1 100 + 0.01 * Day*” – Component density increases at a constant daily rate

“*P1R2C2 if(Day<100, 100, 10)*” – Component density changes on day 100

“*P2R1C1 100 * Day^1.0001^*” – Component density increases at a proportional rate

“*P2R1C2 100 * Day^0.9999^*” – Component density decreases at a proportional rate

“*P2R2C1 0*” – Component is not present in resource

“*P2R2C2 if(Day>=100,10,0)*” – Component is only present in resource from day 100

**Parameters defined**

**Component densities**	Density of each component in each resource. This parameter is the name of the resource and component appended with “Dens” (e.g. “CocklesEnergyDens” is the density of the component called “Energy” in the resource called “Cockles”).

**Parameter 36 – Rule for whether patch results are saved**

Formula to calculated whether patch results are saved at the end of a time step. The formula must return one during time steps in which results are to be saved and zero during time steps in which results are not to be saved. Any previously defined parameters can be used in this calculations (e.g. TimeStep=50 saves patch results during time step 50).

**Parameter type**

Number

**Potential parameters**

Time step, Number of time steps, Time step length. Global variables.

**Header**

*“Rule for whether patch results are saved (1=yes; 0=no):”*

**Examples**

“*0*” – Patch results are not saved

“*if(Time=12,1,0)*” – Patch results are saved when Time = 12

********************** Forager parameters **********************

**Parameter 37 – Number of forager types**

Number of forager types (e.g. species, age classes, sexes) in the simulation.

**Parameter type**

Number >= 1

**Potential parameters**

None

**Header**

*“Number of forager types (>=1):”*

**Examples**

“*1*” – The number of forager types is set to 1

“*2*” – The number of forager types is set to 2

**Parameter 38 – Forager type names**

Name of each forager type. Forager type names must be unique, cannot contain spaces or non-alphanumerical characters, and cannot start with a number.

**Parameter type**

Name for each forager type. The forager type is indicated by “T” followed by the forager type number.

**Potential parameters**

None

**Header**

*“Name of each forager type (text without spaces):”*

**Examples**

“*T1 Oystercatcher*” – Forager type 1 is named “Oystercatcher”

“*T2 Knot*” – Forager type 2 is named “Knot”

**Parameter 39 – Forager type colours**

Colour in which forager type is displayed in the Map Image and Foragers Tab-sheet. The colour is calculated using the rgb formula.

**Parameter type**

Colour for each forager type. The forager type is indicated by “T” followed by the forager type number.

**Potential parameters**

None

**Header**

*“Colour of each forager type (colour code):”*

**Examples**

“*T1 rgb(255,0,0)*” – Forager type 1 is displayed in red

“*T2 rgb(0,0,255)*” – Forager type 2 is displayed in blue

**Parameter 40 – Number of foragers**

Number of foragers in each forager type. This is the total number of foragers that can be present in the model system. The number actually present during any given time step is controlled by immigration (parameter 46) and emigration (parameter 63) of foragers to and from the system, and the number of foragers that die (parameters 71 and 72).

**Parameter type**

Number (>= 0) for each forager type. The forager type is indicated by “T” followed by the forager type number.

**Potential parameters**

None

**Header**

*“Number of foragers of each forager type (>=0):”*

**Examples**

“*T1 10000*” – The initial number of foragers in forager type 1 is set to 10000

“*T2 1000*” – The initial number of foragers in forager type 2 is set to 1000

**Parameter 41 – Number of individuals in foragers**

Number of individuals represented by each forager of each forager type. Each forager represents one or more identical individuals. The facility for foragers to represent more than one individual (i.e. for foragers to be super-individuals) allows MORPH to simulate very large population sizes without incorporating an excessive number of foragers.

**Parameter type**

Number (>= 1) for each forager type. The forager type is indicated by “T” followed by the forager type number.

**Potential parameters**

None

**Header**

*“Number of individuals represented by each forager of each forager type (>=1):”*

**Examples**

“*T1 100*” – Each forager of forager type 1 represents 100 individuals

“*T2 1*”– Each forager of forager type 1 represents 1 individual

**Parameters defined**

**ForagerNumber**	Number of forager.

**Parameter 42 – Number of forager constants**

Number of forager constants calculated for each forager. The values of forager constants are calculated for each forager at the start of a simulation and remain constant throughout a simulation.

**Parameter type**

Number (>= 0)

**Potential parameters**

None

**Header**

*“Number of forager constants (>=0):”*

**Examples**

“*0*” – The number of forager constants is set to zero

“*2*” – The number of forager constants is set to 2

**Parameter 43 – Forager constant names**

Name of each forager constant. Forager constant names must be unique, cannot contain spaces or non-alphanumerical characters, and cannot start with a number.

**Parameter type**

Name for each forager constant. The forager constant is indicated by “C” followed by the forager constant number.

**Potential parameters**

None

**Header**

*“Name of each forager constant (text without spaces):”*

**Examples**

“*C1 Efficiency*” – Forager constant 1 is named “Efficiency”

“*C2 Dominance*” – Forager constant 2 is named “Dominance”

**Parameters defined**

**Forager constants**	Value of each forager constant for each forager. This parameter has the same name as the forager constant to which it refers.

**Parameter 44 – Forager constant values**

Formula to calculate the value of each forager constant at the start of a simulation. The formula is calculated once for each forager (e.g. using Uniform(0, 1) to calculate a forager constant generates a uniform random number, meaning that each will have a different value of the forager constant). The formula can include any previous forager constants (e.g. the second forager constant can depend on the values of the first forager constant).

**Parameter type**

Formula for each forager type and forager constant. The forager type is indicated by “T” followed by the forager type number, and the forager constant by “C” followed by the forager constant number.

**Potential parameters**

Forager number. Previously defined forager constants.

**Header**

*“Value of each forager constant for each forager type (formula):”*

**Examples**

“*T1C1 Normal(1, 0.1, 0, 2)*”

– Forager constant from normal distribution with mean = 1, sd = 0.1, min = 0 and max =2

“*T1C2 Uniform(0, 1)*” – Forager constant from uniform distribution between 0 and 1

“*T2C1 1*” – Forager constant set to 1

“*T2C2 ForagerNumber*” – Forager constant set to forager number

**Parameters defined**

**Forager counts**	Two parameters to count the number of foragers on a patch matching certain criteria.

(1) *Count by forager type*. Number of individuals of a specified forager type on a patch. Named as “Count” appended with the forager type name (e.g. “CountOystercatcher” is the number of individuals of the forager type named “Oystercatcher”).

(2) *Count by forager constant*. Number of individuals with the same forager constant value as a focal forager (i.e. the forager being processed by MORPH). Named as “CountSame” appened with the forager constant name (e.g. “CountSameDominance” is the number of individuals with the same dominance as the focal individual).

**Forager constant ranks**	Two parameters to rank a focal forager with other foragers on a patch.

(1) *Rank by forager constant and group by forager type*. Number of individuals of a specified forager type on a patch with a lower value of a specified forager constant. Named as “Rank” appended with the forager constant name, appended with the forager type name (e.g. “RankDominanceOystercatcher” is the number of individual Oystercatchers with a lower Dominance than the focal forager).

(2) *Rank by forager constant and group by forager constant*. Number of individuals with the same value of a specified forager constant which have a lower value of another specified forager constant. Named by as “Rank” appended with the name of the forager constant to rank by, appended by “Same”, appended by the forager constant to group by (e.g. “RankDominenceSameEfficiency” is the number of individuals with the same efficiency as the focal forager which have a lower dominance).

**Parameter 45 – Forager ordering expression**

Formula to calculate an ordering value for each forager at the start of each time step. The foragers are then processed according to their ordering value with the those with the highest values processed first. The formula can generate different values on different time steps and so foragers can be processed in a different order in different time steps (e.g. Uniform(0,1) will generate a uniform random number for each forager during each time step, meaning that foragers are processed in a random order).

**Parameter type**

Formula

**Potential parameters**

Time step, Number of time steps, Time step length. Global variables. Forager number. Forager constants.

**Header**

*“Expression to order processing of foragers during each time step (formula):”*

**Examples**

“*Uniform(0, 1)*” – Foragers are processed in a random order during each time step

“*Dominance*” – Foragers are processed in order of increasing dominance

**Parameter 46 – Immigration probability**

Formula to calculate the probability that a forager will immigrate into the model system. All foragers are external to the system at the start of a simulation. Each time step a random uniform number between 0 and 1 is calculated for each forager and the forager immigrates if this number is less than their probability of immigration. Foragers always immigrate during the first time step if their probability of immigration is one.

**Parameter type**

Formula for each forager type. The forager type is indicated by “T” followed by the forager type number.

**Potential parameters**

Time step, Number of time steps, Time step length. Global variables. Forager number. Forager constants.

**Header**

*“Probability of immigration to the system for each forager type (formula):”*

**Examples**

“*T1 1*”

– Probability of immigration is one, and so all foragers immigrate to the system during the first time step

“*T2 if(Day=50,1,0)*” – All foragers immigrate to the system on day 50

**Parameter 47 – Initial resource component store sizes**

Formula to calculate the total amount of a resource component within a forager when the forager immigrates to the system. The amount of resource component is measured in the same units as the density of resource component in resources (e.g. if resource component density in resources is measured in g per unit of resource, the amount of a resource component within foragers is measured in g). The formula is calculated when the forager immigrates to the system.

**Parameter type**

Formula for each forager type and resource component. The forager type is indicated by “T” followed by the forager type number and the resource component by “C” followed by the resource component number.

**Potential parameters**

Time step, Number of time steps, Time step length. Global variables. Forager number. Forager constants.

**Header**

*“Initial size of each component store of immigrant foragers of each forager type (formula):”*

**Examples**

“*T1C1 100*” – Initial resource component store size is 100

“*T1C2 10*” – Initial resource component store size is 100

“*T2C1 100 + 0.1 * Day*” – Initial resource component size increases through time

“*T2C2 100 – 0.1 * Day*” – Initial resource component size decreases through time

**Parameter 48 – Target resource component store sizes**

Formula to calculate the target amount of a resource component for a forager during each time step. Foragers attempt to meet this target by adjusting the proportion of time spent feeding. The amount of resource component is measured in the same units as the density of resource components in resources (e.g. if resource component density in resources is measured in g per unit of resource, the amount of a resource component within foragers is measured in g).

**Parameter type**

Formula for each forager type and resource component. The forager type is indicated by “T” followed by the forager type number and the resource component by “C” followed by the resource component number.

**Potential parameters**

Time step, Number of time steps, Time step length. Global variables. Forager number. Forager constants.

**Header**

*“Target size of each component store for each forager type (formula):”*

**Examples**

“*T1C1 100*” – Target resource component store size is 100

“*T1C2 10*” – Target resource component store size is 100

“*T2C1 100 + 0.1 * Day*” – Target resource component size increases through time

“*T2C2 100 – 0.1 * Day*” – Target resource component size decreases through time

**Parameters defined**

**Initial store size**	Size of each component store at start of time step. Named as the component name appended with “InitStore” (e.g. “EnergyInitStore” is the size of the store of the component named “Energy” at the start of a time step).

**Target store size**	Target size of each component store. Named as the component name appended with “TargetStore” (e.g. “EnergyTargetStore” is the target store size of the component named “Energy”).

**Parameter 49 – Memory time**

Duration of time over which foragers remember their past foraging success and metabolism. Time is measured in the same unit as the duration of time steps. MORPH displays an error message if the time does not equal a whole number of time steps.

**Parameter type**

Number for each forager type. The forager type is indicated by “T” followed by the forager type number.

**Potential parameters**

None

**Header**

*“Memory time for each forager type (whole number of time steps):”*

**Examples**

“*T1 24*” – Forager type 1 remembers foraging success and metabolism over 24 time units

“*T2 10*” – Forager type 2 remembers foraging success and metabolism over 10 time units

**Parameter 50 – Number of diets**

Number of diets consumed by foragers. A diet is a collection of resources.

**Parameter type**

Number

**Potential parameters**

None

**Header**

*“Number of diets (>=1):”*

**Examples**

“*1*” – Number of diets is set to 1

“*5*” – Number of diets is set to 5

**Parameter 51 – Diet names**

Name of each diet. Diet names must be unique, cannot contain spaces or non-alphanumerical characters, and cannot start with a number.

**Parameter type**

Name for each diet. The diet is indicated by “D” followed by the diet number.

**Potential parameters**

None

**Header**

*“Name of each diet (text without spaces):”*

**Examples**

“*D1 CocklesDiet*” – Diet 1 is named “CocklesDiet”

“*D2 MusselsDiet*” – Diet 2 is named “MusselsDiet”

**Parameter 52 – Number of resources in diets**

Number of resources contained in each diet.

**Parameter type**

Number for each diet. The diet is indicated by “D” followed by the diet number.

**Potential parameters**

None

**Header**

*“Number of resources in each diet (>=1):”*

**Examples**

“*D1 1*” – Diet 1 contains 1 resource

“*D2 2*” – Diet 2 contains 2 resources

**Parameter 53 – Names of resources in diets**

Name of each resource in each diet. These must be names of resources specified earlier in the parameter file. MORPH displays an error message if an unknown resource name is found, or if a duplicate resource names are found within the same diet.

**Parameter type**

Name for each diet and resource. The diet is indicated by “D” followed by the diet number and the resource by “R” followed by the resource number.

**Potential parameters**

None

**Header**

*“Name of each resource in each diet (resource name):”*

**Examples**

“*D1R1 Cockles*” – Diet 1 contains cockles

“*D2R1 SmallMussels*” – Diet 2 contains small and large musssels

“*D2R2 LargeMussels*”

**Parameters defined**

**Diet density**	Density of each diet on each patch. This is the total density of all resources within the diet. This parameter is the name of the diet appended with “Dens” (e.g. “CocklesDens” is the density of a diet called “Cockles”).

**Diet component density**	Density of each component in each diet. This is the average component density across all resources within a diet, weighted by the density of each resource. This parameter is the name of the diet and component appended with “Dens” (e.g. “CocklesEnergyDens” is the density of the component called “Energy” in the diet called “Cockles”).

**Parameter 54 – Number of forager variables**

Number of forager variables calculated for each forager. The values of forager variables are calculated for each forager at the start of each time step and so can change throughout a simulation.

**Parameter type**

Number (>= 0)

**Potential parameters**

None

**Header**

*“Number of forager variables (>=0):”*

**Examples**

“*0*” – The number of forager variables is set to zero

“*2*” – The number of forager variables is set to 2

**Parameter 55 – Forager variable names**

Name of each forager variable. Forager variable names must be unique, cannot contain spaces or non-alphanumerical characters, and cannot start with a number.

**Parameter type**

Name for each forager variable. The forager variable is indicated by “V” followed by the forager variable number.

**Potential parameters**

None

**Header**

*“Name of each forager variable (text without spaces):”*

**Examples**

“*V1 NumParasite*” – Forager variable 1 is named “NumParasite”

“*V2 Moulting*” – Forager variable 1 is named “Moulting”

**Parameters defined**

Forager variables	Value of each forager variable for each forager. This parameter has the same name as the forager variable to which it refers.

**Parameter 56 – Forager variable values**

Formula to calculate the value of each forager variable at the start of each time step. The formula is calculated for each forager at the start of each time step (e.g. using Uniform(0, 1) to calculate a forager variable generates a uniform random number for each forager during each time step, meaning that each will have a different value of the forager variable during each time step). The formula can include any previous forager variables (e.g. the second forager variable can depend on the values of the first forager variable).

**Parameter type**

Formula for each forager variable. The forager variable is indicated by “V” followed by the forager variable number.

**Potential parameters**

Time step, Number of time steps, Time step length. Global variables. Patch number, Patch size, Patch location. Patch variables. Resource densities, Resource component densities, Diet densities, Diet component densities. Forager number. Forager constants. Forager counts. Forager constant ranks. Target store size, Initial store size. Previously defined forager variables.

**Header**

*“Value of each forager variable for each forager type (formula):”*

**Examples**

“*T1V1 Uniform(0,100)*” – Forager variable drawn from a uniform distribution between 0 and 1000

“*T1V2 if(Day<100, 0, 1)*” – Forager variable is 0 before day 100 and 1 afterwards

“*T2V1 Efficiency*” – Forager variable is set to same value as previously defined forager constant

“*T2V2 Dominance*” – Forager variable is set to same value as previously defined forager constant

**Parameters defined**

**Forager variable ranks**	Two parameters to rank a focal forager with other foragers on a patch.

(1) *Rank by forager variable and group by forager type*. Number of individuals of a specified forager type on a patch with a lower value of a specified forager variable. Named as “Rank” appended with the forager variable name, appended with the forager type name (e.g. “RankNumParasiteOystercatcher” is the number of individual Oystercatchers with a lower number of parasites than the focal forager).

(2) *Rank by forager variable and group by forager constant*. Number of individuals with the same value of a specified forager constant which have a lower value of a specified forager variable. Named by as “Rank” appended with the name of the forager variable to rank by, appended by “Same”, appended by the forager constant to group by (e.g. “RankNumParasiteSameEfficiency” is the number of individuals with the same efficiency as the focal forager which have a lower number of parasites).

**Parameter 57– Diet consumption rate**

Formula to calculate the amount of each diet that a forager can consume per unit time. The units of diet consumption depend on the units of resource density and time (e.g. if resource density is measured as resource items per unit size, resource consumption is measured in resource units per unit time; if resource density is measured as resource mass per unit size, resource consumption is measured in resource mass per unit time). Time is measured in the same unit as the duration of time steps.

**Parameter type**

Formula for each forager type and diet. The forager type is indicated by “T” followed by the forager type number and the diet by “D” followed by the diet number.

**Potential parameters**

Time step, Number of time steps, Time step length. Global variables. Patch number, Patch size, Patch location. Patch variables. Resource densities, Resource component densities, Diet densities, Diet component densities. Forager number. Forager constants. Forager counts. Forager constant ranks. Target store size, Initial store size. Forager variables. Forager variable ranks.

**Header**

*“Rate of consuming each diet for each forager type (formula):”*

**Examples**

“*T1D1 10*” – Diet consumption rate is 10

“*T1D2 0.1 * CocklesDietDens*”

– Diet consumption rate increases with increased cockle diet density

“*T2D1 0*” – Diet consumption rate is zero (diet is not consumed)

“*T2D2 10 – 0.1 * (CountKnot / PatchSize)*”

– Diet consumption rate decreases with increased knot density

**Parameter 58 – Maximum diet consumption rate**

Formula to calculate the maximum amount of each diet that a forager can consume per unit time. The units of diet consumption depend on the units of resource density and time (e.g. if resource density is measured as resource items per unit size, resource consumption is measured in resource units per unit time; if resource density is measured as resource mass per unit size, resource consumption is measured in resource mass per unit time). Time is measured in the same unit as the duration of time steps.

**Parameter type**

Formula for each forager type and diet. The forager type is indicated by “T” followed by the forager type number and the diet by “D” followed by the diet number.

**Potential parameters**

Time step, Number of time steps, Time step length. Global variables. Patch number, Patch size, Patch location. Patch variables. Resource densities, Resource component densities, Diet densities, Diet component densities. Forager number. Forager constants. Forager counts. Forager constant ranks. Target store size, Initial store size. Forager variables. Forager variable ranks.

**Header**

*“Maximum rate of consuming each diet for each forager type (formula):”*

**Examples**

“*T1D1 10*” – Maximum consumption rate set to 10

“*T1D2 10 + 0.01 * Day*” – Maximum consumption rate increases through time

“*T2D1 10 – 0.01 * Day*” – Maximum consumption rate decreases through time

“*T2D2 0*” – Maximum consumption rate is zero

**Parameter 59 – Assimilation efficiency**

Formula to calculate the proportion of the total amount of resource component consumed that is assimilated into the forager’s system (e.g. a value of 0.5 means that 50% of the consumed resource component is assimilated into the forager’s system).

**Parameter type**

Formula for each forager type, diet and resource component. for each forager type and diet. The forager type is indicated by “T” followed by the forager type number, the diet by “D” followed by the diet number and the resource component by “C” followed by the resource component number.

**Potential parameters**

Time step, Number of time steps, Time step length. Global variables. Patch number, Patch size, Patch location. Patch variables. Resource densities, Resource component densities, Diet densities, Diet component densities. Forager number. Forager constants. Forager counts. Forager constant ranks. Target store size, Initial store size. Forager variables. Forager variable ranks.

**Header**

*“Efficiency of assimilating each component from each diet for each forager type (formula):”*

**Examples**

“T1D1C1 0.5” – Assimilation efficiency is set to 0.5

“T1D1C2 0.5 – 0.001 * Day” – Assimilation efficiency decreases through time

“T1D2C1 0.5 + 0.001 * Day” – Assimilation efficiency increases through time

“T1D2C2 0.01 * CocklesDietEnergyDens”

– Assimilation efficiency depends on the energy density of cockles

“T2D1C1 0.01 * EnergyInitStore”

– Assimilation efficiency depends on the energy store size at the start of time step

“T2D1C2 0.01 * EnergyTargetStore”

– Assimilation efficiency depends on the energy store target size

“T2D2C1 0.5 * Efficiency” – Assimilation efficiency depends on a forager’s efficiency

“T2D2C2 0.5 * Dominance” – Assimilation efficiency depends on a forager’s dominance

**Parameter 60 – Feeding metabolic rate**

Formula to calculate the amount of a resource component that is lost from the forager’s body per unit time while feeding. The amount of resource component is measured in the same units as the density of resource component in resources (e.g. if resource component density in resources is measured in g per unit of resource, the amount of a resource component within foragers is measured in g). Time is measured in the same unit as the duration of time steps.

**Parameter type**

Formula for each forager type and resource component. The forager type is indicated by “T” followed by the forager type number and the resource component by “C” followed by the resource component number.

**Potential parameters**

Time step, Number of time steps, Time step length. Global variables. Patch number, Patch size, Patch location. Patch variables. Resource densities, Resource component densities, Diet densities, Diet component densities. Forager number. Forager constants. Forager counts. Forager constant ranks. Target store size, Initial store size. Forager variables. Forager variable ranks.

**Header**

*“Rate of metabolising each component while feeding on each diet for each forager type (formula):”*

**Examples**

“*T1C1 10*” – Metabolic rate is constant

“*T1C2 10 + 0.1 * (Temperature – 10)*” – Metabolic rate increases with decreased temperature

“*T2C1 10 + 0.01 * Day*” – Metabolic rate increases through time

“*T2C2 10 + 0.1 * EnergyInitStore*” – Metabolic rate increases with increased energy store size

**Parameter 61 – Resting metabolic rate**

Formula to calculate the amount of a resource component that is lost from the forager’s body per unit time while resting. The amount of resource component is measured in the same units as the density of resource component in resources (e.g. if resource component density in resources is measured in g per unit of resource, the amount of a resource component within foragers is measured in g). Time is measured in the same unit as the duration of time steps.

**Parameter type**

Formula for each forager type and resource component. The forager type is indicated by “T” followed by the forager type number and the resource component by “C” followed by the resource component number.

**Potential parameters**

Time step, Number of time steps, Time step length. Global variables. Patch number, Patch size, Patch location. Patch variables. Resource densities, Resource component densities, Diet densities, Diet component densities. Forager number. Forager constants. Forager counts. Forager constant ranks. Target store size, Initial store size. Forager variables. Forager variable ranks.

**Header**

*“Rate of metabolising each component while resting for each forager type (formula):”*

**Examples**

“*T1C1 10*” – Metabolic rate is constant

“*T1C2 10 + 0.1 * (Temperature – 10)*” – Metabolic rate increases with decreased temperature

“*T2C1 10 + 0.01 * Day*” – Metabolic rate increases through time

“*T2C2 10 + 0.1 * EnergyInitStore*” – Metabolic rate increases with increased energy store size

**Parameter 62 – Moving metabolic rate**

Formula to calculate the amount of a resource component that is lost from the forager’s body per unit time while moving between patches. The amount of resource component is measured in the same units as the density of resource component in resources (e.g. if resource component density in resources is measured in g per unit of resource, the amount of a resource component within foragers is measured in g). Time is measured in the same unit as the duration of time steps.

**Parameter type**

Formula for each forager type and resource component. The forager type is indicated by “T” followed by the forager type number and the resource component by “C” followed by the resource component number.

**Potential parameters**

Time step, Number of time steps, Time step length. Global variables. Forager number. Forager constants. Target store size, Initial store size.

**Header**

*“Rate of metabolising each component while moving for each forager type (formula):”*

**Examples**

“*T1C1 10*” – Metabolic rate is constant

“*T1C2 10 + 0.1 * EnergyInitStore*” – Metabolic rate increases with increased energy store size

“*T2C1 10 + 0.01 * Day*” – Metabolic rate increases through time

“*T2C2 10 – 0.01 * Day*” – Metabolic rate decreases through time

**Parameter 63 – Emigration fitness measure**

Formula to calculate the probability that a forager will emigrate from the model system. Each time step a random uniform number between 0 and 1 is calculated for each forager and the forager emigrates if this number is less than their probability of emigration.

**Parameter type**

Formula for each forager type. The forager type is indicated by “T” followed by the forager type number.

**Potential parameters**

Time step, Number of time steps, Time step length. Global variables. Patch number, Patch size, Patch location. Patch variables. Resource densities, Resource component densities, Diet densities, Diet component densities. Forager number. Forager constants. Forager counts. Forager constant ranks. Target store size, Initial store size. Forager variables. Forager variable ranks.

**Header**

*“Expected fitness measure of emigration for each forager type (formula):”*

**Examples**

“*T1 0*” – Expected fitness measure of emigration is zero

“*T2 if(Day>100, 1, 0)*” – Expected fitness measure of emigration is one after day 100

**Parameters defined**

**Patch comparison**	Comparison of the values of a patch variable on the current and target patches. This is the difference in patch variable values (i.e. the value of the patch variable on the target patch minus the value on the current patch). Named as “Compare” appended with the patch variable name (e.g. “CompareWaterDepth” stores the difference in values of the patch variable named “WaterDepth”).

**Movement distance**	Number of distance units between the current and target patches. Named as “MoveDist”.

**Parameter 64 – Movement times**

Formula to calculate the duration of time taken to move between patches. Time is measured in the same unit as the duration of time steps. MORPH displays an error message if the time does not equal a whole number of time steps.

**Parameter type**

Formula for each forager type. The forager type is indicated by “T” followed by the forager type number.

**Potential parameters**

Time step, Number of time steps, Time step length. Global variables. Forager number. Forager constants. Target store size, Initial store size. Patch comparison. Movement distance.

**Header**

*“Time to move between patches for each forager type (whole number of time steps):”*

**Examples**

“*T1 if(MoveDist > 100, 1, 0)*” – Movement takes one time unit if patches are more than 100 units apart

“*T2 0*” – Movement between patches takes no time

**Parameters defined**

**Movement time**	Time required to move between the current and target patches. Named as “MoveTime”.

**Movement cost**	Amount of each component metabolised while moving between the current and target patches. Named as the component name appended with “MoveMetab” (e.g. “EnergyMoveMetab” is the amount of the component named “Energy” metabolised while moving between the current and target patches.

**Parameter 65 – Patch location rules**

Formula to calculate whether a patch can be located by a forager. A forager can only move to a patch which it can locate. The formula must return one for patches which can be located and zero for patches which cannot be located. MORPH displays an error message if a value other than one or zero is calculated.

**Parameter type**

Formula for each forager type. The forager type is indicated by “T” followed by the forager type number.

**Potential parameters**

Time step, Number of time steps, Time step length. Global variables. Patch number, Patch size, Patch location. Patch variables. Resource densities, Resource component densities, Diet densities, Diet component densities. Forager number. Forager constants. Forager counts. Forager constant ranks. Target store size, Initial store size. Forager variables. Forager variable ranks. Patch comparison. Movement distance, Movement time, Movement cost.

**Header**

*“Rule for whether patch can be located for each forager type (1=yes; 0=no):”*

**Examples**

“*T1 if(MoveDist < 100, 1, 0)*” – Foragers can only locate patches less than 100 distance units apart

“*T2 if(WaterDepth = 0, 1, 0)*” – Foragers can only locate patches that are not covered with water

**Parameter 66 – Fitness assessment rules**

Formula to calculate whether fitness measures can be assessed on a patch. The formula must return one for patches on which fitness can be assessed and zero for patches on which fitness cannot be assessed. MORPH displays an error message if a value other than one or zero is calculated.

**Parameter type**

Formula for each forager type. The forager type is indicated by “T” followed by the forager type number.

**Potential parameters**

Time step, Number of time steps, Time step length. Global variables. Patch number, Patch size, Patch location. Patch variables. Resource densities, Resource component densities, Diet densities, Diet component densities. Forager number. Forager constants. Forager counts. Forager constant ranks. Target store size, Initial store size. Forager variables. Forager variable ranks. Patch comparison. Movement distance, Movement time, Movement cost.

**Header**

*“Rule for whether fitness measure can be assessed for each forager type (1=yes; 0=no):”*

**Examples**

“*T1 if(MoveDist < 100, 1, 0)*”

– Foragers can only assess fitness on patches less than 100 distance units apart

“*T2 if(WaterDepth = 0, 1, 0)*”

– Foragers can only assess fitness on patches that are not covered with water

**Parameter 67 – Expected fitness measures**

Formula to calculate the expected fitness measure on patches on which fitness measures cannot be assessed.

**Parameter type**

Formula for each forager type. The forager type is indicated by “T” followed by the forager type number.

**Potential parameters**

Time step, Number of time steps, Time step length. Global variables. Patch number, Patch size, Patch location. Patch variables. Resource densities, Resource component densities, Diet densities, Diet component densities. Forager number. Forager constants. Forager counts. Forager constant ranks. Target store size, Initial store size. Forager variables. Forager variable ranks. Patch comparison. Movement distance, Movement time, Movement cost.

**Header**

*“Expected fitness measure on patches on which fitness cannot be assessed for each forager type (formula):”*

**Examples**

“*T1 0.1 * EnergyInitStore*”

– Expected fitness measure depends on energy store size at start of time step

“*T2 0.001 * MoveDist*”

– Expected fitness measure depends on distance between patches

**Parameters defined**

**Feeding effort**	Proportion of time spent feeding during a time step. Named as “PropTimeFeeding”.

**Consumption**	Three parameters are defined to store details of diet consumption.

(1) Rate of consuming diet (number of diet units consumed per unit time). Named as “DietConsRate”.

(2) Rate of consuming components from diet (number of component units consumed per unit time). Named as the component named appended with “ConsRate” (e.g. “EnergyConsRate” is the rate of consuming the component named “Energy”.

(3) Rate of assimilating components from diet (number of component units assimilated per unit time). Named as the component named appended with “AssimRate” (e.g. “EnergyAssimRate” is the rate of assimilating the component named “Energy”.

**Metabolism**	Metabolic rate. Named as the component name appended with “MetabRate” (e.g. “EnergyMetabRate” is the overall metabolic rate of the component named “Energy”).

**Past consumption**	Three parameters are defined to store details of past diet consumption.

(1) Past rate of consuming diet (average number of diet units consumed per unit time over previous time steps). Named as “PastDietConsRate”.

(2) Past rate of consuming components from diet (average number of component units consumed per unit time over previous time steps). Named as the component named appended with “PastConsRate” (e.g. “EnergyPastConsRate” is the past rate of consuming the component named “Energy”.

(3) Past rate of assimilating components from diet (average number of component units assimilated per unit time over previous time steps). Named as the component named appended with “PastAssimRate” (e.g. “EnergyPastAssimRate” is the past rate of assimilating the component named “Energy”.

**Past metabolism**	Past rate of metabolising components (average number of component units metabolised per unit time over previous time steps). Named as the component named appended with “PastMetabRate” (e.g. “EnergyPastMetabRate” is the past rate of metabolising the component named “Energy”.

**Final store size**	Size of each component store at end of time step. Named as the component name appended with “FinalStore” (e.g. “EnergyFinalStore” is the size of the store of the component named “Energy” at the end of a time step).

**Parameter 68 – Number of fitness components**

Number of fitness components used by foragers.

**Parameter type**

Number (>= 1)

**Potential parameters**

None

**Header**

*“Number of fitness components (>=1):”*

**Examples**

“1” – Number of fitness components is set to 1

“2” – Number of fitness components is set to 2

**Parameter 69 – Fitness component names**

Name of each fitness component. Fitness component names must be unique, cannot contain spaces or non-alphanumerical characters, and cannot start with a number.

**Parameter type**

Name for each fitness component. The fitness component is indicated by “C” followed by the fitness component number.

**Potential parameters**

None

**Header**

*“Name of each fitness component (text without spaces):”*

**Examples**

“*C1 Starvation*” – Fitness measure 1 is named “Starvation”

“*C2 Predated*” – Fitness measure 2 is named “Predated”

**Parameter 70 – Fitness measure values**

Formula to calculate each fitness measure for each forager type. MORPH displays an error message if the calculated value is less than zero. Fitness measures are calculated for each fitness component and then multiplied together to obtain the combined fitness measure. Foragers move to patches and select diets with the maximum combined fitness measure.

**Parameter type**

Formula for each forager type and fitness component. The forager type is indicated by “T” followed by the forager type number and the fitness component by “C” followed by the fitness component number.

**Potential parameters**

Time step, Number of time steps, Time step length. Global variables. Patch number, Patch size, Patch location. Patch variables. Resource densities, Resource component densities, Diet densities, Diet component densities. Forager number. Forager constants. Forager counts. Forager constant ranks. Target store size, Initial store size. Forager variables. Forager variable ranks. Patch comparison. Movement distance, Movement time, Movement cost. Feeding effort. Consumption. Metabolism. Past consumption, Past metabolism. Final store size.

**Header**

*“Fitness measure for each fitness component for each forager type (formula):”*

**Examples**

“*T1C1 EnergyAssimRate*” – Fitness measure increases with increased energy assimilation rate

“*T1C2 if(WaterDepth > 0, 0, 1)*” – Fitness measure is zero if water depth is greater than zero

“*T2C1 EnergyFinalStore*” – Fitness measure increases with increased final energy store size

“*T2C2 DietConsRate*” – Fitness measure increases with increased diet consumption rate

**Parameter 71 – Feeding and resting survival probability**

Formula to calculate the probability of surviving each fitness component while on a patch. MORPH displays an error message if the probability is not between zero and one. Survival probabilities are calculated for each fitness measure and the multiplied together to obtain the combined survival probability.

**Parameter type**

Formula for each forager type and fitness component. The forager type is indicated by “T” followed by the forager type number and the fitness component by “C” followed by the fitness component number.

**Potential parameters**

Time step, Number of time steps, Time step length. Global variables. Patch number, Patch size, Patch location. Patch variables. Resource densities, Resource component densities, Diet densities, Diet component densities. Forager number. Forager constants. Forager counts. Forager constant ranks. Target store size, Initial store size. Forager variables. Forager variable ranks. Patch comparison. Movement distance, Movement time, Movement cost. Feeding effort. Consumption. Metabolism. Past consumption, Past metabolism. Final store size.

**Header**

*“Survival probability while feeding and resting for each fitness component for each forager type (formula):”*

**Examples**

“*T1C1 if(EnergyFinalStore>0, 1, 0)*” – Forager dies if energy store size is zero at end of time step

“*T1C2 0.999*” – Forager has a constant chance of dying each time step

“*T2C1 1*” – Forager has a survival probability of 1

“*T2C2 if(Day>100, 0.99, 1)*” – Forager has a higher mortality rate after day 100

**Parameter 72 – Moving survival probability**

Formula to calculate the probability of surviving each fitness component while moving between patches. MORPH displays an error message if the probability is not between zero and one. Survival probabilities are calculated for each fitness measure and the multiplied together to obtain the combined survival probability.

**Parameter type**

Formula for each forager type and fitness component. The forager type is indicated by “T” followed by the forager type number and the fitness component by “C” followed by the fitness component number.

**Potential parameters**

Time step, Number of time steps, Time step length. Global variables. Forager number. Forager constants. Target store size, Initial store size. Patch comparison. Movement distance, Movement time, Movement cost. Final store size.

**Header**

*“Survival probability while moving for each fitness component for each forager type (formula):”*

**Examples**

“*T1C1 if(EnergyFinalStore>0, 1, 0)*” – Forager dies if energy store size is zero at end of time step

“*T1C2 0.999*” – Forager has a constant chance of dying each time step

“*T2C1 1*” – Forager has a survival probability of 1

“*T2C2 if(Day>100, 0.99, 1)*” – Forager has a higher mortality rate after day 100

**Parameter 73 – Rule for whether forager results are saved**

Formula to calculate whether forager results are saved at the end of a time step. The formula must return one during time steps in which results are to be saved and zero during time steps in which results are not to be saved. Any previously defined parameters can be used in this calculations (e.g. TimeStep=50 saves forager results during time step 50).

**Parameter type**

Number

**Potential parameters**

Time step, Number of time steps, Time step length. Global variables.

**Header**

*“Rule for whether forager results are saved (1=yes; 0=no):”*

**Examples**

“*0* “– Forager results are not saved

“*1*” – Forager results are saved during each time step

# 7. Example parameter file

This section describes the example parameter file (EXAMPLE.PAR). Section 3 describes how to run the example parameter file. The example file describes a system incorporating two patches, “Patch1” and “Patch2”, each containing two resources, “SmallFood” and “LargeFood”. Patch 1 contains more resources but is only available during daylight. Resources contain two resource components, “Energy” and “Toxin”. The large food contains more energy but also contains toxin, whereas the small food contains less energy but no toxin. Two forager types are present, “RiskMin” and “RateMax”. The risk-minimising forager type behaves in ways that minimise its exposure to risks (i.e. consuming the toxin), whereas the rate-maximising forager type behaves in ways that maximise its rate of assimilating energy, irrespective of whether this exposes it to more risks. In the following sections, each set of parameters are listed in *italics* and “quotes”, followed by a description of the parameters. These sections should be read with the model running or paused.

“********************* GENERAL PARAMETERS *********************

*Name of simulation (text):*

*Example*

*Name of map background image file (file name or blank line):*

*Background.bmp*

*Name of map foreground image file (file name or blank line):*

*Foreground.bmp*

*Minimum coordinates of map image (coordinates):*

*0 0*

*Maximum coordinates of map image (coordinates):*

*0.5 1*

*Relative size of parameter and expression memory (>=1):*

*1*”

The simulation is named, map background and foreground image files identified, the size of the map image defined and the size of the parameter and expression memory set to the default value. The background image is black and is drawn behind the patches and foragers. The foreground image displays two white squares drawn around the boundaries of the two patches.

“********************* GLOBAL PARAMETERS *********************

*Number of time steps (>=1):*

*200*

*Duration of each time step (>0):*

*6*”

The number of time steps is set to 200 and the duration of each time step is set to 6. In this example, the unit of time is assumed to be hours and so the 50 days (200 x 6 hours) will be simulated. This time unit must now be used throughout the parameter file.

“*Number of global variables (>=0):*

*3*

*Name of each global variable (text without spaces):*

*V1 Day*

*V2 Time*

*V3 Daylight*

*Value of each global variable (formula or file):*

*V1 (TimeStepLength*(TimeStep-1)) div 24*

*V2 (TimeStepLength*(TimeStep-1)) mod 24*

*V3 if((Time>6) and (Time<=18),1,0)*

*Colour of each global variable (colour code):*

*V1 rgb(0,0,128)*

*V2 rgb(0,0,128)*

*V3 rgb(0,0,128)*

*Rule for whether global results are saved (1=yes; 0=no):*

*1*”

Three global variables, “Day”, “Time” and “Daylight” are defined and formulae given to calculate their values. These global variables are displayed in dark blue. Global results are saved during each time step. The values of global variables can be seen on the *Variables* or *Details* tab-sheets. The variables “Day”, “Time” and “Daylight” are now available to be used elsewhere in the parameter file.

“********************* PATCH PARAMETERS *********************

*Number of patches (>=1):*

*2*

*Name of each patch (text without spaces):*

*P1 Patch1*

*P2 Patch2*

*Coordinate system (1=X/Y; 2=latitude/longitude):*

*1*

*Size of each patch (>0):*

*P1 100000*

*P2 100000*

*Central coordinates of each patch (coordinates):*

*P1 0 0*

*P2 0 100*”

Two patches, “Patch1” and “Patch2”, are defined, and the coordinate system assumed to be X/Y. Both patches have an area of 100000. In this example, the unit of distance is assumed to be m, and so both patches have an area of 100000 m^2^. This unit of distance must now be used throughout the parameter file. The central coordinates of the patches are set to 0, 0m and 0, 100m, and so the patches are 100m apart.

“*Number of map boundaries displayed for each patch (>=1):*

*P1 1*

*P2 1*

*Number of coordinates for each map boundary of each patch (>=3):*

*P1B1 4*

*P2B1 4*

*Coordinates for each map boundary of each patch (coordinates):*

*P1B1L1 0.05 0.55*

*P1B1L2 0.05 0.95*

*P1B1L3 0.45 0.95*

*P1B1L4 0.45 0.55*

*P2B1L1 0.05 0.05*

*P2B1L2 0.05 0.45*

*P2B1L3 0.45 0.45*

*P2B1L4 0.45 0.05”*

The patch boundaries are defined. Both patches are drawn as squares in the map image, with the safe patch above the risky patch.

“*Number of patch variables (>=0):*

*1*

*Name of each patch variable (text without spaces):*

*V1 Available*

*Colour of each patch variable (colour code):*

*V1 rgb(0,0,128)*

*Value of each patch variable on each patch (formula or file):*

*P1V1 if((Daylight=1),1,0)*

*P2V1 1*”

A patch variable named “Available” is defined, and is displayed in dark blue. Available is 1 on patch 1 during daylight (Daylight=1) and zero during night time (Daylight=0). Available is always 1 on patch 2. The values of patch variables can be seen on the *Variables* or *Details* tab-sheets. The variable “Available” is now available to be used elsewhere in the parameter file.

“*Rule for whether patch results are displayed (1=yes; 0=no):*

*1*

*Colour of each patch (colour code):*

*P1 if((Daylight=1),rgb(0,196,0),rgb(0,64,0))*

*P2 if((Daylight=1),rgb(196,0,0),rgb(64,0,0))*”

Patch results are displayed in the *Details*, *Resources* and *Foragers* tab-sheets during each time step. Patch 1 is displayed in bright green during daylight and dark green during night. Patch 2 is displayed in bright red during daylight and dark red during night.

“*Number of resources (>=1):*

*2*

*Name of each resource (text without spaces):*

*R1 SmallFood*

*R2 LargeFood*

*Colour of each resource (colour code):*

*R1 rgb(0,128,0)*

*R2 rgb(128,0,0)*

*Initial density of each resource on each patch (formula):*

*P1R1 1000*

*P1R2 200*

*P2R1 500*

*P2R2 100*

*Expression to update each resource density on each patch (formula):*

*P1R1 SmallFoodDensity*(power(0.5,(1/(NumTimeStep-1))))*

*P1R2 LargeFoodDensity*(power(0.5,(1/(NumTimeStep-1))))*

*P2R1 SmallFoodDensity*(power(0.5,(1/(NumTimeStep-1))))*

*P2R2 LargeFoodDensity*(power(0.5,(1/(NumTimeStep-1))))*”

Two resources are defined, “SmallFood” and “LargeFood”. Small food is displayed in dark green, and large food is displayed in dark red. Initial density food densities are higher on patch 1. Resources can be in any unit (e.g. mass of food, number of individual food items), but the same unit must be used throughout the parameter file. In this example, resource density is assumed to be measured in food items m^-2^. During each time step the densities of resources are reduced by a small amount (in addition to depletion by foragers), such that, in the absence of foragers, resource densities would be 50% of starting values at the end of a simulation.

“*Number of resource components (>=1):*

*2*

*Name of each resource component (text without spaces):*

*C1 Energy*

*C2 Toxin*

*Density of each component in each resource on each patch (formula):*

*P1R1C1 (10*(1-(0.5*(TimeStep/NumTimeStep))))*

*P1R1C2 0*

*P1R2C1 (50*(1-(0.5*(TimeStep/NumTimeStep))))*

*P1R2C2 1*

*P2R1C1 (10*(1-(0.5*(TimeStep/NumTimeStep))))*

*P2R1C2 0*

*P2R2C1 (50*(1-(0.5*(TimeStep/NumTimeStep))))*

*P2R2C2 1”*

Two resource components are defined, “Energy” and “Toxin”. In this example, component densities are measured as the amount of component within an individual food item of each resource (as resource density was measured as food items m^-2^). Components can be measured in any units. In this example Energy is measured in KJ and Toxin in mg. These units must be used consistently throughout the parameter file. Toxin density is assumed to be 1 mg per large food item (resource 2) and zero in small food. It is assumed that the energy of food items decreases by 50% during the course of a simulation.

“*Rule for whether patch results are saved (1=yes; 0=no):*

*1*”

Patch results are saved during all time steps.

“********************* FORAGER PARAMETERS *********************

*Number of forager types (>=1):*

*2*

*Name of each forager type (text without spaces):*

*T1 RiskMin*

*T2 RateMax*

*Colour of each forager type (colour code):*

*T1 rgb(0,255,0)*

*T2 rgb(255,0,0)*

*Number of foragers of each forager type (>=0):*

*T1 50*

*T2 50*

*Number of individuals represented by each forager of each forager type (>=1):*

*T1 10*

*T2 10*”

Two forager types are defined, “RiskMin” and “RateMax”, each comprised of 50 foragers, each representing 10 individuals. The risk-minimising foragers are displayed in bright green, and the rate-maximising foragers displayed in bright red.

“*Number of forager constants (>=0):*

*3*

*Name of each forager constant (text without spaces):*

*C1 Species*

*C2 Efficiency*

*C3 Dominance*

*Value of each forager constant for each forager type (formula):*

*T1C1 1*

*T1C2 Normal(1,0.25,0,10)*

*T1C3 Uniform(0,1)*

*T2C1 1*

*T2C2 Normal(1,0.25,0,10)*

*T2C3 Uniform(0,1)*”

Three forager constants are defined to store a forager’s species, efficiency at finding food and dominance. Both forager types are assumed to be of the same species. Foraging efficiency is drawn from a normal distribution with unit mean and a standard deviation of 0.25. Dominance is drawn from a uniform distribution between 0 and 1. The variables “Species”, “Efficiency” and “Dominance” are now available to be used elsewhere in the parameter file.

“*Expression to order processing of foragers during each time step (formula):*

*Uniform(0,1)*”

Foragers are processed in a random order during each time step.

“*Probability of immigration to the system for each forager type (formula):*

*T1 1*

*T2 1*

*Initial size of each component store of immigrant foragers of each forager type (formula):*

*T1C1 5000*

*T1C2 0*

*T2C1 5000*

*T2C2 0*

*Target size of each component store for each forager type (formula):*

*T1C1 5000*

*T1C2 0*

*T2C1 5000*

*T2C2 0*”

All foragers immigrate to the system during the first time step. The initial size and target size of the toxin store is zero. The initial size and target size of the energy store is 5000 KJ.

“*Memory time for each forager type (whole number of time steps):*

*T1 24*

*T2 24*”

Foragers remember their foraging success and metabolism over the last 24 hours (4 time steps). This parameter is used to calculate average foraging success and metabolism over previous time steps (4 in this example).

“*Number of diets (>=1):*

*2*

*Name of each diet (text without spaces):*

*D1 SmallFoodDiet*

*D2 AllFoodDiet*

*Number of resources in each diet (>=1):*

*D1 1*

*D2 2*

*Name of each resource in each diet (resource name):*

*D1R1 SmallFood*

*D2R1 SmallFood*

*D2R2 LargeFood*”

Two diets are defined. The small food diet contains small food, and the all food diet contains both small and large food.

“*Number of forager variables (>=0):*

*2*

*Name of each forager variable (text without spaces):*

*V1 CompetitorDensity*

*V2 RelativeDominance*

*Value of each forager variable for each forager type (formula):*

*T1V1 (CountRateMax+CountRiskMin)/PatchSize*

*T1V2 RankDominanceSameSpecies/(CountSameSpecies+0.00001)*

*T2V1 (CountRateMax+CountRiskMin)/PatchSize*

*T2V2 RankDominanceSameSpecies/(CountSameSpecies+0.00001)*”

Two forager variables are defined. Competitor density is calculated from the numbers of rate-maximising and risk-minimising foragers divided by patch area. The local dominance of a forager is calculated from its dominance rank relative to foragers of the same species (RankDominanceSameSpecies) (i.e. the number of foragers with lower dominance) divided by the number of foragers of the same species (CountSameSpecies) plus a small number to avoid division by zero errors if no foragers are present on a patch. These parameters could be used to calculate the effect of interference competition on consumption rate, although this is not done in this example.

“*Rate of consuming each diet for each forager type (formula):*

*T1D1 Efficiency*((20*SmallFoodDietDensity)/(50+SmallFoodDietDensity))*

*T1D2 Efficiency*((20*AllFoodDietDensity)/(50+AllFoodDietDensity))*

*T2D1 Efficiency*((20*SmallFoodDietDensity)/(50+SmallFoodDietDensity))*

*T2D2 Efficiency*((20*AllFoodDietDensity)/(50+AllFoodDietDensity))*”

The rate of consuming each diet for a forager of average foraging efficiency is calculated from food density (i.e. (20 * SmallFoodDietDensity) / (50 + SmallFoodDietDensity)). This is then multiplied by a forager’s foraging efficiency to find its particular consumption rate.

“*T1D1 Efficiency*((20*SmallFoodDietDensity)/(50+SmallFoodDietDensity))*

*T1D2 Efficiency*((20*AllFoodDietDensity)/(50+AllFoodDietDensity))*

*T2D1 Efficiency*((20*SmallFoodDietDensity)/(50+SmallFoodDietDensity))*

*T2D2 Efficiency*((20*AllFoodDietDensity)/(50+AllFoodDietDensity))*”

Maximum food consumption is assumed to be the same as food consumption rate.

“*Efficiency of assimilating each component from each diet for each forager type (formula):*

*T1D1C1 0.5*

*T1D1C2 1*

*T1D2C1 0.5*

*T1D2C2 1*

*T2D1C1 0.5*

*T2D1C2 1*

*T2D2C1 0.5*

*T2D2C2 1*”

Assimilation efficiencies of energy and toxin are 0.5 and 1 respectively.

“*Rate of metabolising each component while feeding on each diet for each forager type (formula):*

*T1D1C1 50*

*T1D1C2 0*

*T1D2C1 50*

*T1D2C2 0*

*T2D1C1 50*

*T2D1C2 0*

*T2D2C1 50*

*T2D2C2 0*

*Rate of metabolising each component while resting for each forager type (formula):*

*T1C1 50*

*T1C2 0*

*T2C1 50*

*T2C2 0*

*Rate of metabolising each component while moving for each forager type (formula):*

*T1C1 50*

*T1C2 0*

*T2C1 50*

*T2C2 0*”

Energy is metabolised at a rate of 50 KJ h^-1^. Toxin is not metabolised.

“*Expected fitness measure of emigration for each forager type (formula):*

*T1 0*

*T2 0*

*Time to move between patches for each forager type (whole number of time steps):*

*T1 0*

*T2 0*

*Rule for whether patch can be located for each forager type (1=yes; 0=no):*

*T1 if((Available=1),1,0)*

*T2 if((Available=1),1,0)*

*Rule for whether fitness measure can be assessed for each forager type (1=yes; 0=no):*

*T1 1*

*T2 1*

*Expected fitness measure on patches on which fitness cannot be assessed for each forager type (formula):*

*T1 0*

*T2 0*”

The expected fitness measure of emigration is set to zero and so foragers do not emigrate from the system (i.e. they stay in the system until the end of a simulation or until they die). The time to move between patches is set to zero, and so movement between patches is instantaneous. Patches can only be located when available is 1 on a patch. Fitness measures can be assessed on all patches.

“*Number of fitness components (>=1):*

*2*

*Name of each fitness component (text without spaces):*

*C1 Starvation*

*C2 Poisoning*

*Fitness measure for each fitness component for each forager type (formula):*

*T1C1 EnergyAssimRate*

*T1C2 if((ToxinAssimRate=0),1,0)*

*T2C1 EnergyAssimRate*

*T2C2 1*

*Survival probability while feeding and resting for each fitness component for each forager type (formula):*

*T1C1 if((EnergyFinalStore>0),1,0)*

*T1C2 if((ToxinFinalStore>1000),0.99,1)*

*T2C1 if((EnergyFinalStore>0),1,0)*

*T2C2 if((ToxinFinalStore>1000),0.99,1)*

*Survival probability while moving for each fitness component for each forager type (formula):*

*T1C1 if((EnergyFinalStore>0),1,0)*

*T1C2 if((ToxinFinalStore>1000),0.99,1)*

*T2C1 if((EnergyFinalStore>0),1,0)*

*T2C2 if((ToxinFinalStore>1000),0.99,1)*”

Two fitness measures, “Starvation” and “Poisoning”, are defined. The combined fitness measure for rate-maximising foragers only depends on energy assimilation rate; these foragers always consume the all food diet and occupy the patch which maximises energy assimilation rate. The combined fitness measure of risk-minimising foragers depends on energy assimilation rate and toxin assimilation rate; these foragers feed on the diet and patch which yields maximum energy assimilation, but with the constraint that toxin assimilation rate must be zero. The rate-maximising foragers consume the all food diet, and so consume the toxin. The risk-minimising foragers avoid toxin by consuming the small food diet. Foragers die if their energy reserves fall to zero, if they consume more than 1000 mg of toxin. The rate-maximising foragers have a higher energy assimilation rate, but some die of poisoning by the end of the simulation. The risk-minimising foragers have a lower energy assimilation rate, and some die of starvation by the end of the simulation.

“*Rule for whether forager results are saved (1=yes; 0=no):*

*1*”

Forager results are saved during each time step.

# 7. Structure of result files

MORPH outputs results to the following six text files, with file extensions determined by the *ResFileExt* command line parameter, in a folder determined by *ResPath* command line parameter.

**File name	Description**

Log	General summary of the simulation and the parameter file

Global	Global results

Patch	Patch-specific results

Resource	Resource-specific results

Diet	Diet-specific results

Forager	Forager-specific results

The first file stores information on the model version used, parameter file, result files, duration of the simulation and whether or not the simulation completed successfully. All the remaining files store the predicted results of a simulation and have the same basic structure. They are tab-delimited text files, with their first row storing a list of result names and subsequent rows storing the value of each result. The following section lists the results stored in each file.

# 8. Description of results files

**Log results**

Information on the model version used, parameter file, result files, duration of the simulation and whether or not the simulation completed successfully.

**Global results**

One row output for each time step. The contents of each column are as follows.

Time step number

Time step length – Duration of each time step in time units

Global variables – Value of each global variable

**Patch results**

One row output for each patch during each time step. The contents of each column are as follows.

Time step number

Patch number

Patch name

Patch size – Patch size in area units

Patch location 1 – Patch location measured in either X coordinates or longitude on the globe.

Patch location 2 – Patch location measured in either Y coordinates or latitude on the globe.

Patch variables – Value of each patch variable

Forager numbers – Numbers of each forager type on patch

**Resource results**

One row output for each resource and patch combination during each time step. The contents of each column are as follows.

Time step number

Patch number

Resource number

Resource name

Amount consumed – Number of resource units consumed by foragers during time step

Resource density – Density of resource units at end of time step

Component densities – Density of each component within each resource

**Diet results**

One row output for each diet and patch combination during each time step. The contents of each column are as follows.

Time step number

Patch number

Diet number

Diet name

Diet density – Density of diet units at end of time step

Component densities – Density of each component within each diet

**Forager results**

One row output for each forager during each time step. The contents of each column are as follows.

Time step number

Forager number

Forager type

Forager type name – Name of each forager type

Number immigrating – Number of individuals of each forager type to immigrate into the system

Number present – Number of individuals of each forager type present in the system

Number dead – Number of individuals of each forager type killed by each fitness component

Number emigrated – Number of individuals of each forager type emigrated from the system

Forager constants – Value of each forager constant

Fitness measure – Fitness measure for current patch and diet combination

Movement state – 1 if forager is moving between patches, 0 if forager is on a patch

Current patch – Number of current patch

Forager variables – Value of each forager variable

Current diet – Number of current diet (0 if no diet is being consumed)

Feeding effort – Proportion of time spent feeding

Maximum consumption rate – Maximum diet consumption rate in diet units per time unit

Consumption rate – Actual diet consumption rate in diet units per time unit

Component consumption rate – Rate of consuming each component in component units per time unit

Component assimilation rate – Rate of assimilating each component in component units per time unit

Component metabolism rate – Rate of metabolising each component in component units per time unit

Component target store – Target size of component store in component units

Initial component store – Size of component store at start of time step in component units

Final component store – Size of component store at end of time step in component units
